# The endogenous cannabinoid system gates plasticity of tonic GABA inhibition

**DOI:** 10.1113/JP289183

**Published:** 2026-05-27

**Authors:** Roberto Colangeli, Fiorenzo Conti

**Affiliations:** ^1^ Department of Experimental and Clinical Medicine, Section of Neuroscience and Cell Biology Università Politecnica delle Marche Ancona Italy; ^2^ Center for Neurobiology of Aging IRCCS INRCA Ancona Italy

**Keywords:** endocannabinoid system, neurosteroids, tonic GABA inhibition

## Abstract

**Abstract:**

GABAergic neurotransmission generates two types of inhibition: a phasic inhibition, determined by the transient activation of synaptic GABA_A_ receptors, that elicits inhibitory postsynaptic currents, and a tonic inhibition caused by persistent activation of extrasynaptic GABA_A_ receptors by ‘ambient’ GABA. Changes in the efficacy of GABAergic transmission are important mechanisms contributing to experience‐dependent modifications of brain function. Mechanisms underlying changes in synaptic GABAergic efficacy have been investigated and, among them, endocannabinoid (eCB)‑dependent GABAergic synaptic plasticity is a well‑characterised mechanism. Little, however, is known about the potential control of, for example, eCB signalling on extrasynaptic GABA tone. By using whole‐cell patch‐clamp recordings, we showed that a brief depolarisation of cortical pyramidal neurons is associated with a transient increase in tonic GABA inhibition that is dependent on CB1 receptor activity and eCB mobilisation. In addition, we showed that this depolarisation‐dependent plasticity of tonic inhibition does not arise from the transient increase of ambient GABA concentration but requires intracellular neurosteroid synthesis since the pharmacological inhibition of the P450scc enzyme responsible for the neurosteroid cascade synthesis blunted this phenomenon. These data provide evidence that, under sustained neuronal activity, eCBs and neurosteroids are engaged to finely tune tonic extrasynaptic GABAergic inhibition in activated neurons.

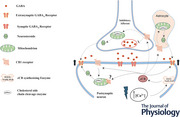

**Key points:**

- Besides phasic synaptic inhibition, GABA mediates a form of tonic extrasynaptic inhibition.
- The physiological mechanisms that tune the tonic GABA inhibition are largely unclear.
- Here we show that brief depolarisation of cortical pyramidal neurons transiently and reversibly potentiates tonic GABA inhibition.
- Increased extracellular GABA concentration or changes in GABA turnover do not account for plasticity of tonic inhibition.
- Endocannabinoid signalling and neurosteroids are required for plasticity of tonic GABA inhibition.

## Graphical abstract

**Abstract figure legend** Postsynaptic depolarisation and increases in intracellular Ca^2^^+^ ([Ca^2^^+^]_i_) trigger the synthesis of endocannabinoids (eCBs), which are hypothesised to enhance tonic GABAergic inhibition via two primary pathways. In the proposed autocrine mechanism, eCBs activate postsynaptic somatodendritic or mitochondrial CB1 receptors, stimulating *de novo* neurosteroidogenesis via the P450scc enzyme; notably, eCBs may also modulate GABA_A_ receptors through direct binding. Alternatively, in the proposed paracrine pathway, eCBs act as retrograde messengers at presynaptic and astrocytic CB1 receptors, triggering the extracellular release of neurosteroids that subsequently act on postsynaptic extrasynaptic GABA_A_ receptors.

## Introduction

Besides the classic phasic synaptic GABAergic transmission, which results from large but transient elevations in GABA concentration in the synaptic cleft and the binding to GABA_A_ receptors in the active zone, GABA activity subserves a form of tonic inhibition determined by the persistent activation of extrasynaptic GABA_A_ receptors by low concentrations of ambient GABA in the extracellular space (Cherubini & Conti, 2001; Farrant & Nusser, 2005; Mody & Pearce, 2004; Semyanov et al., 2003, 2004). A large body of evidence has demonstrated that extrasynaptic GABAergic tone plays an important role in many physiological processes, for example cognitive functions (Lee et al., 2016), sensory processing (Kwak et al., 2020), stress response (Liu et al., 2014) and emotional behaviour (Botta et al., 2015). Moreover, aberrant tonic GABA inhibition may play a role in addiction (Anstee et al., 2013), memory deficits (Zurek et al., 2014), postpartum depression (Maguire & Mody, 2008) and epilepsy (Cope et al., 2009). Despite the important roles played by tonic GABAergic inhibition in brain functions, there is little information on the mechanisms by which this form of inhibition can change its strength to adapt to external stimuli. Reorganisation of extrasynaptic GABA_A_ receptors or modulation of ambient GABA concentration through changes of GABA spillover or reuptake may account for long‐term adaptations of tonic inhibition (Bragina et al., 2008; Conti et al., 2004). In addition, neuromodulators, such as neurosteroids, endocannabinoids (eCBs) and dopamine can directly act on extrasynaptic GABA_A_ receptors to alter the strength of tonic GABA inhibition (Bakas et al., 2017; Hoerbelt et al., 2015; Stell et al., 2003).

GABAergic synaptic transmission exhibits activity‐dependent changes in its efficacy that provide flexibility to neural circuits (Kullmann et al., 2012). The molecular mechanisms by which synaptic GABAergic plasticity occurs have been largely characterised (Castillo et al., 2011; Kullmann et al., 2012) and, among them, eCB‐mediated GABA synaptic plasticity appears to be a widespread phenomenon across the brain (Chevaleyre et al., 2006; Kano et al., 2009). The eCB system encompasses the two main endogenous cannabinoids, anandamide (AEA) and 2‐arachidonoyl glycerol (2‐AG), cannabinoid (CB) receptors and the molecular machinery responsible for the mobilisation of eCBs. eCB‐mediated plasticity of inhibitory transmission requires the synthesis of eCBs in postsynaptic neurons following elevated intracellular calcium influx, and the activation of the type 1 cannabinoid (CB1) receptor expressed presynaptically at inhibitory terminals (Colangeli et al., 2021; Kano et al., 2009). Activation of the CB1 receptor results in transient or long‐lasting depression of synaptic GABAergic transmission (Chevaleyre & Castillo, 2003; Colangeli et al., 2023; Tanimura et al., 2010). In addition to the axon terminals, CB1 receptors are also expressed in astrocytes (Navarrete & Araque, 2008), and in mitochondrial membranes where they also contribute to neuronal transmission and plasticity (Hebert‐Chatelain et al., 2016).

On these grounds, we investigated whether, besides its well‐known role in modulating synaptic GABAergic transmission, the eCB system may contribute to the fine tuning of tonic GABA inhibition. We identified a cascade of events in which a brief depolarisation of somatosensory cortical neurons engages intracellular eCB and neurosteroid signalling to transiently and reversibly enhance tonic GABA inhibition.

## Material and methods

### Animals and ethical approval

Adult (2–3 months) male and female C57BL/6J mice (Charles River, Calco, Italy) were used. Four to six animals per cage were housed under standard environmental conditions (room temperature, 20–22°C; relative humidity, 50–55%; 12 h light–dark cycle with the light phase starting at 6 a.m.) with food and water available *ad libitum* and basic environmental enrichment. All procedures were conducted in accordance with European Union legislation (Directive 2010/63/EU) and were approved by the Italian Ministry of Health (number 40A31.N.ZUK). The well‐being of the animals was continuously monitored.

### Brain slice preparation

Mice of either sex were decapitated under deep anaesthesia with 5% isoflurane inhalation, and the brain was quickly excised for slicing as described previously (Colangeli et al., 2020; Gom et al., 2024). Briefly, coronal brain slices (300 µm) of the first somatic sensory cortex (SI) were prepared with a slicer (model VT1200S, Leica, Wetzlar, Germany) in ice‐cold slicing solution containing the following (in mM): 87 NaCl, 2.5 KCl, 25 NaHCO_3_, 2.5 CaCl_2_, 7 MgCl_2_, 1.25 NaH_2_PO_4_, 25 d‐glucose and 75 sucrose, saturated with 95% O_2_/ 5% CO_2_. Slices were incubated in a holding chamber with oxygenated ACSF containing the following (in mM): 126 NaCl, 2.5 KCl, 2.5 CaCl_2_, 1.5 MgCl_2_, 1.25 NaH_2_PO_4_, 26 NaHCO_3_ and 10 d‐glucose, constantly bubbled with 95% O_2_/5% CO_2_ at pH 7.4 for 30 min at 32°C. Slices were then incubated for at least 45 min in regular ACSF or ACSF supplemented with drugs that required a pre‐incubation period. After incubation, slices were placed in the recording chamber and continuously superfused with regular ACSF at a flow rate of 1.5 mL/min. All experiments were performed at room temperature (22–25°C).

### Drug application

Drugs were stored in frozen aliquots and diluted to the desired concentration in 250 mL ACSF immediately before each experiment. Bicuculline methiodide (BIC; 20 µM), carbachol (CCh; 5 µM), GABA (2 µM), SR‑95531 (gabazine, GBZ; 100 µM) and AP5 (40 µM) were dissolved in ACSF. The 2‑AG synthesis inhibitor DO34 (1 µM), the CB1 receptor antagonist AM251 (10 µM), the GAT‑1 inhibitor NO711 (20 µM), the neurosteroid synthesis inhibitor aminoglutethimide (AMG; 300 µM), allopregnanolone (ALLO; 1 µM), the neutral CB1 receptor antagonist NESS0327 (NESS; 0.5 µM) and DNQX (20 µM) were prepared as concentrated stock solutions in 100% DMSO. Across all experiments, drugs dissolved in DMSO were diluted into 250 mL of recording ACSF to obtain the desired final concentrations. Depending on the volume of stock solution required, the final DMSO content in the bath ranged from approximately 0.04% v/v to a maximum of 0.19% v/v. Control ACSF solutions were supplemented with the same DMSO concentration used in the corresponding drug condition to ensure proper vehicle matching. DMSO, BIC, CCh, GABA, SR‑95531 (GBZ), DNQX, DO34, NO711, NESS0327 and AMG were purchased from Sigma‑Aldrich (Merck, St Louis, MO, USA). AP5, AM251 and ALLO were obtained from Tocris (Bio‑Techne, Abingdon, UK).

### Electrophysiological recordings and data analysis

**Figure 1:**
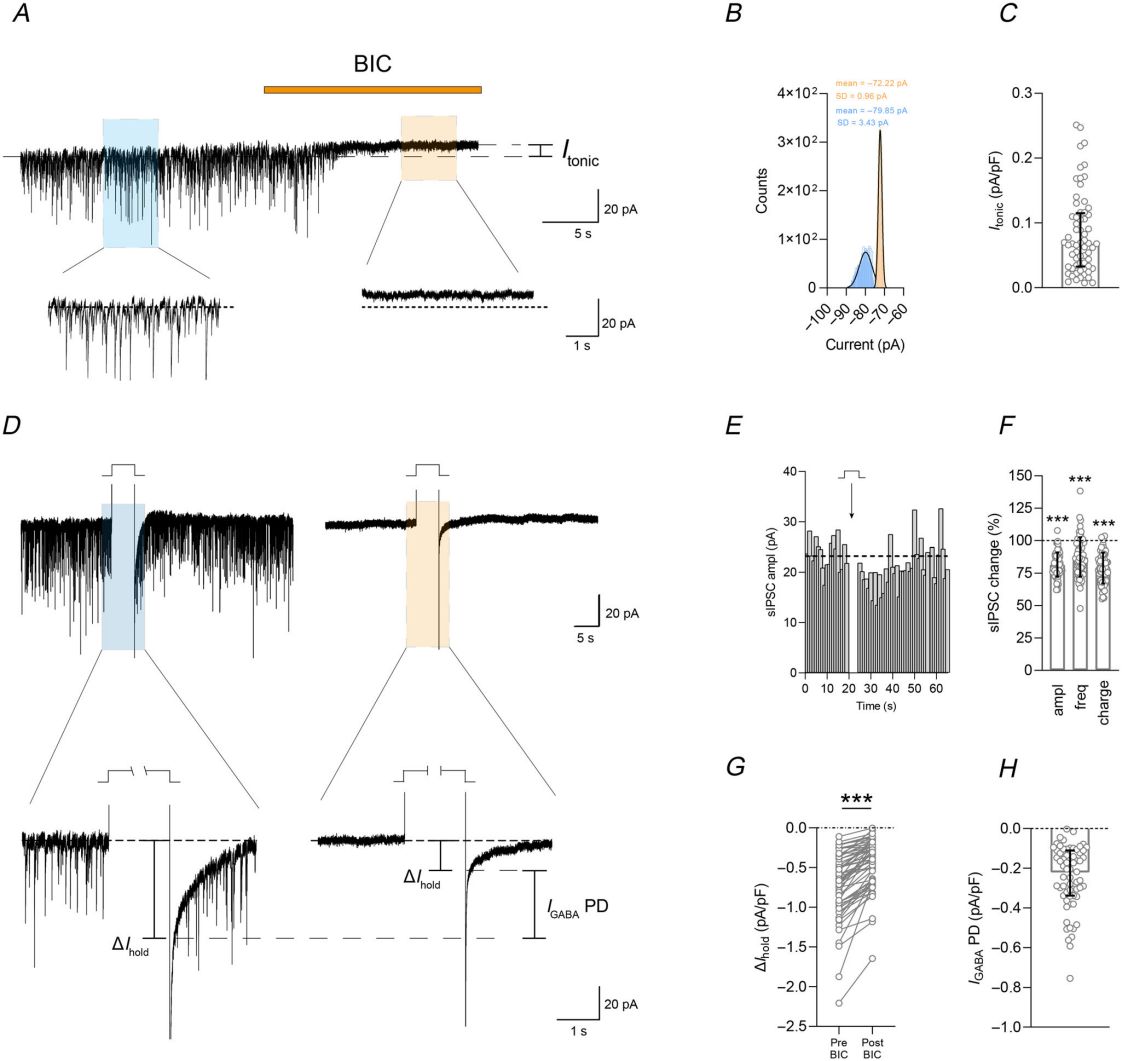
Basal and depolarisation‐dependent tonic GABA inhibition in cortical pyramidal neurons *A*, representative full‐trace recording of tonic and synaptic GABAergic currents in mouse cortical pyramidal neurons. Bath application of the GABA_A_ receptor antagonist bicuculline (BIC, 20 µM; orange bar) abolished spontaneous IPSCs and produced an outward shift in the holding current, reflecting BIC‑sensitive basal tonic current (*I*_tonic_). Below, 5 s segments of the same traces are shown at an expanded timescale, illustrating the holding current during control (cyan) and BIC (orange) conditions. Dashed lines in each inset indicate the baseline (pre‐BIC) holding current level to facilitate visual *I*_tonic_. *B*, current distributions (5 s epochs sampled in 10 ms segments every 100 ms) were fitted with Gaussian functions to extract the mean holding current before (cyan) and after BIC (orange) application. Embedded text shows the mean ± SD of each distribution from the recording shown in *A*. *I*_tonic_ was estimated as the difference between the two Gaussian peaks. *C*, bar graph showing *I*_tonic_, normalised to the cell capacitance (pA/pF). *D*, representative continuous full‑trace recordings obtained before and after the depolarisation step (truncated) during ACSF (pre‐BIC; left) and BIC superfusion (right). In the pre‑BIC condition, spontaneous IPSCs were used to quantify depolarisation‑induced suppression of inhibition (DSI), whereas in the post‑BIC trace synaptic events were abolished. Below, expanded timescale segments corresponding to the cyan (pre‐BIC) and orange (post‐BIC) epochs illustrate 2 s of baseline activity followed by 2 s after the depolarising step. The depolarising step is truncated, with only 0.5 s after its onset and 0.5 s before its offset visible (bracket symbols), to better resolve the pre‐ and post‐depolarisation holding current. Dashed lines are shared between the left and right insets to allow direct visual comparison of Δ*I*_hold_ magnitude; they indicate the initial baseline current and the peak Δ*I*_hold_ level, taken 80 ms after the end of the pulse. *I*_GABA_ PD denotes the BIC‐sensitive component used to compute the depolarisation‐induced tonic current. *E*, sIPSC amplitude (pA) plotted within 1 s time windows from the pre‐BIC trace shown in *D*. The dashed line indicates the mean sIPSC amplitude during the baseline period. *F*, quantification of the depolarisation‐induced reduction in sIPSCs, expressed as percentage change in amplitude (ampl), frequency (freq) and charge transfer (charge) within 10 s windows starting at the DSI peak (or 2 s after pulse termination) and expressed as percentage variation relative to a 10 s baseline period preceding the depolarising step. The dashed line at 100% indicates the pre‐depolarisation baseline. ***P < 0.001 *vs*. 100% baseline; one‑sample *t* test. *G*, paired graph showing Δ*I*_hold_ (pA/pF) before (Pre‐BIC) and after (Post‐BIC) BIC application. The depolarisation‐induced downward shift of the holding current was significantly attenuated in the presence of BIC (***P < 0.001; Wilcoxon test). *H*, bar graph showing the BIC‐sensitive component of the depolarisation‐induced Δ*I*_hold_ (*I*_GABA_ PD), calculated and normalised to cell capacitance (pA/pF). Data in *C*, *F* and *H* are expressed as mean ± SD; each circle represents an individual recorded cell.

Whole‑cell patch‑clamp recordings were obtained from layer 2/3 pyramidal neurons of SI, visually identified under IR‑DIC video microscopy (Zeiss Axioscope FS2, 40× water‑immersion objective) based on their characteristic pyramidal‑shaped soma and the presence of a clearly visible apical dendrite. Tonic GABA currents (*I*_tonic_) were recorded using borosilicate glass pipettes (3–5 MΩ) filled with a high concentration of caesium chloride, so that GABA_A_ receptor‐mediated currents at −70 mV were inward, and most potassium currents were blocked. The pipette solution contained the following (in mM): 130 CsCl, 1 EGTA, 10 HEPES, 4.6 MgCl_2_, 0.1 CaCl_2_, 5 QX‐314, 0.3 Na‐GTP and 4 Mg‐ATP, at pH 7.3 adjusted with CsOH. Some experiments included the neurosteroid synthesis inhibitor AMG (300 µM) in the internal solution. Cells were voltage‐clamped at −70 mV and recordings were performed in the continuous presence of 40 µM d‐AP‐5 [d(–)‐2‐amino‐5‐phosphonopentanoic acid] and 20 µM DNQX (6,7‐dinitroquinoxaline‐2,3‐dione) to block glutamatergic transmission. Basal tonic GABA currents (*I*_tonic_) were analysed as an outward shift in the holding current (*I*_hold_) after bath application of either 20 µM BIC (Fig. 1*A*) or 100 µM GBZ (Fig. 9*A*). The mean *I*_hold_ was calculated from 5 s recordings by averaging 10 ms segments taken every 100 ms. Synaptic events were excluded by omitting points that fell on IPSCs (Cope et al., 2005, 2009). The resulting clean baseline was quantified by Gaussian fitting of the amplitude histograms generated before and after antagonist application (Fig. 1*B* and Fig. 9*B*). The mean values from the Gaussian fits after antagonist administration were then subtracted from the same baseline pre‐antagonist period to obtain the basal tonic GABA current (*I*_tonic_): *I*_tonic_ = *I*_hold post‐BIC_ ‐ *I*_hold pre‐BIC_ (Fig. 1*C*) and *I*_tonic_ = *I*_hold post‐GBZ_ ‐ *I*_hold pre‐GBZ_ (Fig. 9*C*) normalised to the whole‐cell membrane capacitance (*C*_m_).

To reveal a putative change in the strength of tonic GABAergic inhibition, a depolarisation step was applied to the recorded neuron and the downward shift of the *I*_hold_ calculated at peak taken 80 ms after the end of the depolarisation step (Δ*I*_hold_) was subtracted from the baseline mean *I*_hold_ during the 5 s pre‐depolarisation epoch (Fig. 1*D*). The neuronal depolarisation was chosen to mimic sustained neuronal activity and consisted of holding the cell at 0 mV for 5 s (Colangeli et al., 2023; Yoshida et al., 2002).

To isolate the GABA component of the Δ*I*_hold_ (*I*_GABA_ PD), the depolarisation step was again applied in the continuous presence of BIC (or GBZ) and then the sensitive component of the Δ*I*_hold_ to the antagonist was analysed by subtracting the Δ*I*_hold_ post‐BIC (or post‐GBZ) from the Δ*I*_hold_ pre‐BIC (or pre‐GBZ) as follows: *I*_GABA_PD = Δ*I*_hold post‐BIC_‐Δ*I*_hold pre‐BIC_ (Fig. 1*E*–*F*) and *I*_GABA_PD = Δ*I*_hold post‐GBZ_‐Δ*I*_hold pre‐GBZ_ (Fig. 9*E*–*F*). Both *I*_tonic_ and *I*_GABA_ PD current densities were normalised to *C*_m_. *C*_m_ was obtained in voltage‐clamp mode using a 10 mV depolarising test pulse delivered from a holding potential of −70 mV. *C*_m_ was calculated from the capacitive transient using the Membrane Test function implemented in pClamp (Molecular Devices, Sunnyvale, CA, USA). *C*_m_ was assessed at the beginning and at the end of each recording to ensure recording stability. Recordings were accepted for analysis if either *C*_m_ and access resistance (initial value <30 MΩ) did not change by >20%. The pCLAMP 9 software (Molecular Devices) was used for data analysis. For depolarisation‑induced suppression of inhibition (DSI) experiments, spontaneous IPSCs (sIPSCs) were detected from continuous recordings using Mini Analysis 6.0.7 software (Synaptosoft, Decatur, GA, USA) with a detection threshold set at five times the RMS noise. sIPSC amplitude, frequency and charge were calculated within 1 s time windows. For statistical analysis, a 10 s baseline control period preceding the depolarisation step (consisting of 10 consecutive 1 s bins) was compared to a 10 s interval starting either at the DSI peak or 2 s after the termination of the pulse (Bodor et al., 2005). Recordings were acquired via an Axopatch 200B amplifier (Molecular Devices) filtered at 1 kHz, digitised at 10 kHz and stored on a personal computer.

### Experimental design and statistical analysis

**Figure 2:**
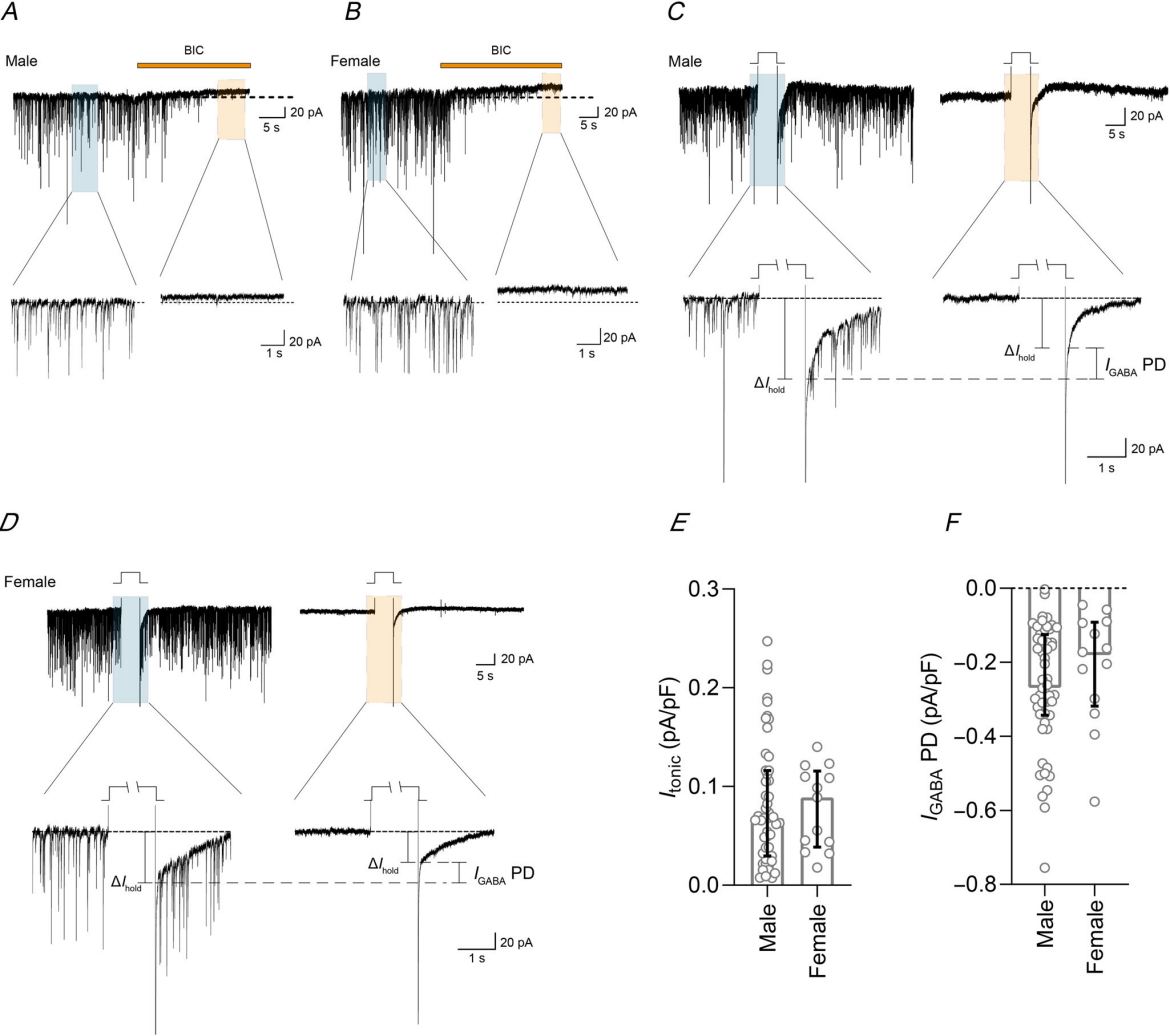
Basal and activity‐dependent tonic GABA inhibition in male and female mice *A* and *B*, representative full‐trace recordings from cortical pyramidal neurons of male (*A*) and female (*B*) mice recorded under control conditions and during BIC application (orange bar). Below each full‐trace, 5 s segments corresponding to the cyan (pre‑BIC) and orange (post‑BIC) epochs are shown at an expanded timescale. Dashed lines in each inset indicate the baseline (pre‐BIC) *I*_hold_ to facilitate visual comparison. *C* and *D*, representative continuous full‑trace recordings from cortical pyramidal neurons of male (*C*) and female (*D*) mice obtained during ACSF (control; left) and BIC superfusion (right), each showing the response to a depolarising step (truncated). Below each full‐trace, expanded segments corresponding to the cyan (pre‐BIC) and orange (BIC) epochs illustrate 2 s of baseline activity followed by 2 s after the depolarising step. The depolarising step is truncated, with only its onset and offset visible (bracket symbols). Dashed lines are shared between the left and right insets to allow direct visual comparison of Δ*I*_hold_ magnitude; they indicate the initial baseline *I*_hold_ and the peak Δ*I*_hold_ level. *I*_GABA_ PD denotes the BIC‐sensitive component used to compute the depolarisation‐induced tonic current. *E*, quantification of the BIC‑sensitive basal tonic current (*I*_tonic_) in cortical pyramidal neurons from male and female mice, revealing no significant sex‑dependent differences. *F*, quantification of the BIC‑sensitive, depolarisation‑evoked tonic current (*I*_GABA_ PD) normalised to cell capacitance in male and female neurons, showing no significant differences between sexes. Data are expressed as mean ± SD, with each circle representing a single cell recorded.

Data obtained from both male and female animals were combined because there were no differences or trends between the sexes (see Fig. 2 in the Results). Each value plotted in the graphs refers to one cell in a separate brain slice. GraphPad Prism version 7 (GraphPad Software Inc., San Diego, CA, USA) was used to perform data analysis. The data were checked for normal distribution (Shapiro–Wilk test). Comparisons among normally distributed data were performed using unpaired or paired Student's *t* tests or one‐way ANOVA followed by Tukey's *post hoc* test for multiple comparisons, when appropriate. When data were not normally distributed, the non‐parametric Wilcoxon signed‐rank test was used for paired samples and the Mann–Whitney *U* test for unpaired samples. When comparisons of non‐normally distributed data were made among more than two groups, the Kruskal–Wallis test followed by Dunn's multiple comparisons test was performed. Data with unbalanced sample sizes and significant variance heterogeneity, as determined by the Brown–Forsythe test, were analysed using Welch's ANOVA followed by Dunnett's T3 *post hoc* test. Differences were considered statistically significant at *P* < 0.05. Data are expressed as mean ± SD.

## Results

### Brief depolarisation of cortical pyramidal neurons transiently enhances tonic GABA inhibition

We first assessed whether in our experimental conditions layer 2/3 cortical pyramidal neurons of SI displayed basal tonic GABA current. We found a small but consistent upward shift of the holding current (*I*_tonic_) of 6.07 ± 3.73 pA [*n*_(cells)_ = 64; *N*_(mice)_ = 35] following BIC application (Fig. 1*A*–*C*). We then investigated whether a physiological mechanism capable of finely tuning the GABA tone during sustained neuronal activity does exist. Since a brief depolarisation of the postsynaptic neuron triggers a transient suppression of synaptic GABA release (DSI) in many brain regions, including the neocortex (Bodor et al., 2005; Colangeli et al., 2023; Földy et al., 2006; Yoshida et al., 2002), we tested whether neuronal depolarisation might also affect extrasynaptic GABAergic tone. We first assessed the presence and magnitude of DSI in layer 2/3 cortical pyramidal neurons and found that a 5 s depolarisation step (from –70 to 0 mV; Fig. 1*D*) applied to the recorded neuron caused a reduction in sIPSC amplitude (81.68 ± 9.39%; *P* < 0.001, one‑sample *t* test), sIPSC frequency (87.41 ± 15.10%; *P* < 0.001, one‑sample *t* test) and sIPSC charge (78.90 ± 11.89%; *P* < 0.001, one‑sample *t* test; Fig. 1*E*–*F*). Having established that DSI was present under our experimental conditions, we next examined the effect of the depolarisation step on the extrasynaptic GABAergic tone. We found a transient downward shift in *I*_hold_ recorded immediately after the end of the depolarisation step (Δ*I*_hold_; Fig. 1*D*). When the depolarisation step was applied again in the continuous presence of BIC, Δ*I*_hold_ was significantly decreased (*P* < 0.001, Wilcoxon test; Fig. 1*G*), but not totally abolished, indicating that the change in *I*_hold_ induced by the sustained neuronal activity is, at least in part, mediated by GABA_A_ receptors. We then quantified the GABA_A_ receptor component of the depolarisation‐induced increase in Δ*I*_hold_ (*I*_GABA_ PD) by subtracting the *I*_hold_ obtained before and after BIC application [−20.52 ± 15.46 pA; *n*_(cells)_ = 64; *N*_(mice)_ = 35] and then normalising the current value to *C*_m_ (Fig. 1*H*).

### Depolarisation‐induced enhancement of tonic GABA inhibition is sex independent

Tonic GABAergic inhibition is known to be sex dependent (Maguire & Mody, 2007; Maguire et al., 2005; Melón et al., 2017; Urban‐Ciecko & Mozrzymas, 2011); we therefore investigated whether either basal or activity‐dependent changes in tonic GABAergic inhibition differed between male and female cortical neurons. To this end, we tested both *I*_tonic_ (Fig. 2*A*–*B*) and *I*_GABA_ PD (Fig. 2*C*–*D*) in pyramidal neurons obtained from SI of male and female mice. BIC application provoked a similar upward shift in *I*_hold_ in both male [*n*_(cells)_ = 51; *N*_(mice)_ = 28] and female [*n*_(cells)_ = 13; *N*_(mice)_ = 7] mice, providing evidence that *I*_tonic_ does not depend on sex (Mann–Whitney test; *P* = 0.704; Fig. 2*E*). We then tested *I*_GABA_ PD and found that the BIC‐sensitive component of the downward Δ*I*_hold_ showed no difference between the sexes (Mann–Whitney test; *P* = 0.305; Fig. 2*F*).

### Depolarisation‐induced potentiation of tonic GABA inhibition is eCB dependent

**Figure 3:**
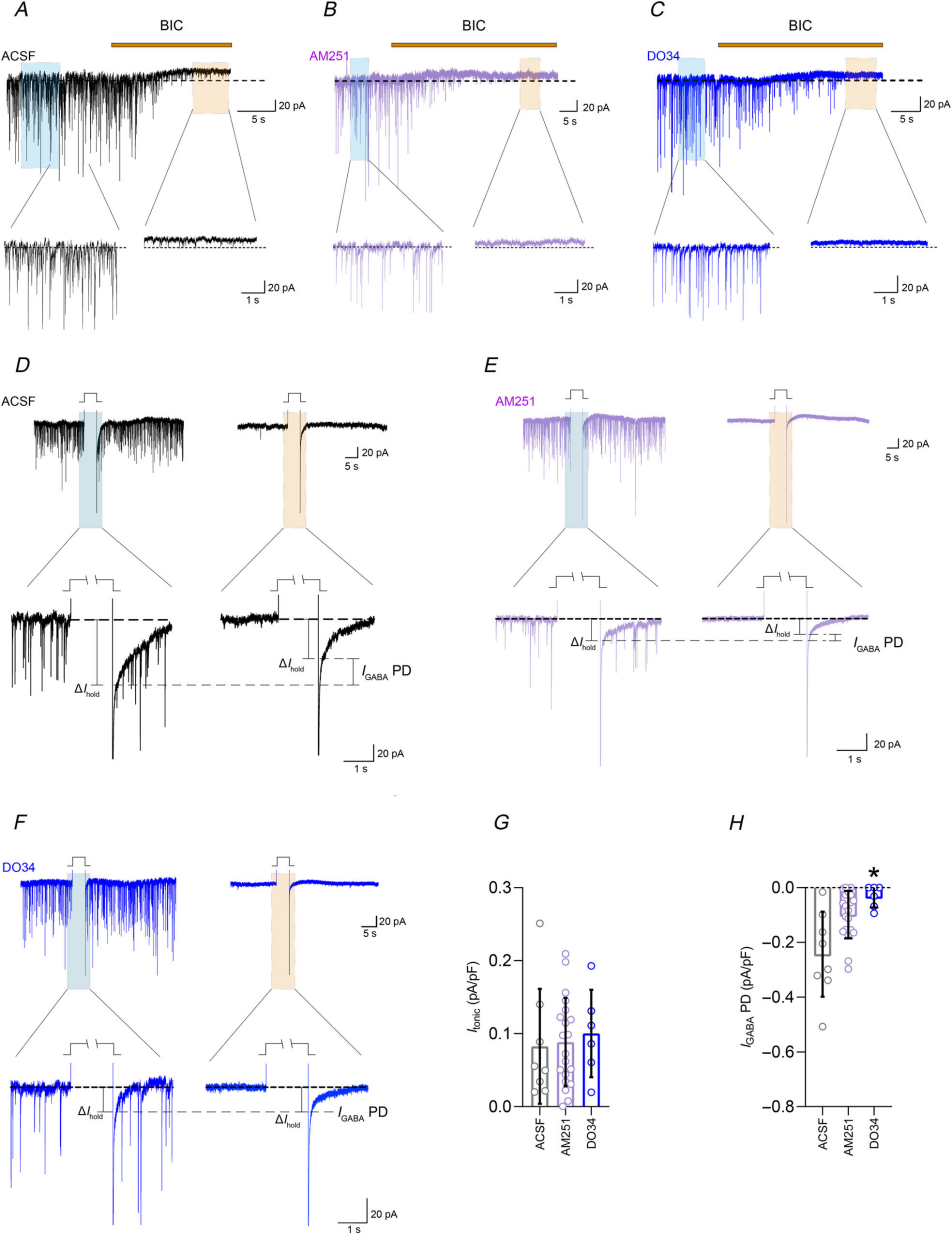
Depolarisation‐induced enhancement of tonic GABA inhibition is eCB signalling dependent *A*–*C*, representative current traces recorded from cortical pyramidal neurons in ACSF (*A*), in the continuous presence of the CB1 receptor antagonist AM251 (10 µM; *B*) and in neurons from slices pre‐incubated with the DAGL inhibitor DO34 (1 µM; *C*). BIC application (20 µM; orange bar) abolished spontaneous IPSCs and produced a comparable outward shift in the holding current (*I*_tonic_) across all conditions. Below each full‐trace, 5 s segments corresponding to the cyan (pre‐BIC) and orange (post‐BIC) epochs are shown at an expanded timescale. Dashed lines in each inset indicate the baseline (pre‐BIC) *I*_hold_ level to facilitate visual comparison. *D*–*F*, representative continuous full‐trace recordings from ACSF‐ (*D*), AM251‐ (*E*) and DO34‐ (*F*) treated slices, obtained during pre‐BIC (left) and BIC (right) superfusion, each showing the response to a depolarising step (truncated). Below each full‐trace, expanded segments corresponding to the cyan (pre‐BIC) and orange (BIC) epochs illustrate 2 s of baseline activity followed by 2 s after the depolarising step. The depolarising step is truncated, with only 0.5 s after its onset and before its offset visible (bracket symbols). Dashed lines are shared between the left and right insets to allow direct visual comparison of Δ*I*_hold_ magnitude; they indicate the initial *I*_hold_ and the peak Δ*I*_hold_ level. *I*_GABA_ PD denotes the BIC‐sensitive component used to compute the depolarisation‐induced tonic current. *G*, summary data showing that the BIC‑sensitive tonic current (*I*_tonic_) does not differ across ACSF, AM251 and DO34 conditions. *H*, summary data showing that inhibition of 2‑AG synthesis (DO34), but not CB1 receptor blockade (AM251), significantly reduces *I*_GABA_ PD. *P = 0.012 ACSF *vs*. DO34; Dunnett's T3 multiple comparisons test. Data are expressed as mean ± SD with each circle representing a single cell recorded.

eCB signalling influences GABA transmission predominantly by activating presynaptic CB1 receptors (Chevaleyre et al., 2006; Colangeli et al., 2023; Kano et al., 2009). From postsynaptic neurons, eCBs can be either constitutively released, or released ‘on demand’ in response to phasic neuronal activation, thus subserving tonic or phasic control of GABA synaptic transmission, respectively (Katona & Freund, 2012). We first assessed whether tonic eCB signalling affects extrasynaptic GABAergic tone. If there is a constitutive action of eCB signalling on tonic GABA, we should be able to unmask this effect by blocking the CB1 receptor or the enzyme responsible for the synthesis of 2‐AG. To this end we recorded *I*_tonic_ in the presence of ACSF only [*n*_(cells)_ = 8; *N*_(mice)_ = 3; Fig. 3*A*], the CB1 antagonist AM251 [*n*_(cells)_ = 20; *N*_(mice)_ = 11; Fig. 3*B*] or the Diacylglycerol lipase (DAGL) enzyme inhibitor DO34 [*n*_(cells)_ = 6; *N*_(mice)_ = 4; Fig. 3*C*]. However, neither AM251 nor DO34 had any effect on *I*_tonic_, since BIC application provoked a similar upward shift of *I*_hold_ in all conditions tested [Kruskal–Wallis test; *H*_(2)_ = 0.508; *P* = 0.775; Fig. 3*G*]. This observation suggests that either eCBs are not constitutively released in the SI cortex or tonic eCB signalling does not affect basal extrasynaptic GABAergic activity. We then asked whether phasic eCB signalling contributes to the depolarisation‐induced potentiation of tonic GABA. We therefore applied a depolarisation step to the postsynaptic neuron, and recorded Δ*I*_hold_ before and after BIC administration in the presence of ACSF only [*n*_(cells)_ = 8; *N*_(mice)_ = 3; Fig. 3*D*], AM251 [*n*_(cells)_ = 20; *N*_(mice)_ = 11; Fig. 3*E*] and DO34 [*n*_(cells)_ = 6; *N*_(mice)_ = 4; Fig. 3*F*]. We then compared *I*_GABA_ PD in the three experimental conditions (Fig. 3*H*) and found that the inhibition of 2‐AG synthesis, but not the blockade of CB1 receptor, significantly reduced *I*_GABA_ PD (Welch's ANOVA; *F*_2,14.15_ _=_ 8.292; *P* = 0.004). Indeed, Dunnett's T3 multiple comparisons test showed a significant effect for 2‐AG synthesis blockade (*P = 0.012 ACSF *vs*. DO34) while CB1 receptor blockade did not reach a statistically significant difference compared to the control group (Dunnett's T3 multiple comparisons test; *P* = 0.066 ACSF *vs*. AM251). These observations indicate that 2‐AG signalling is required for the depolarisation‐induced potentiation of GABA tone.

### CB1 receptor activation contributes to the depolarisation‐induced potentiation of tonic GABAergic inhibition

**Figure 4:**
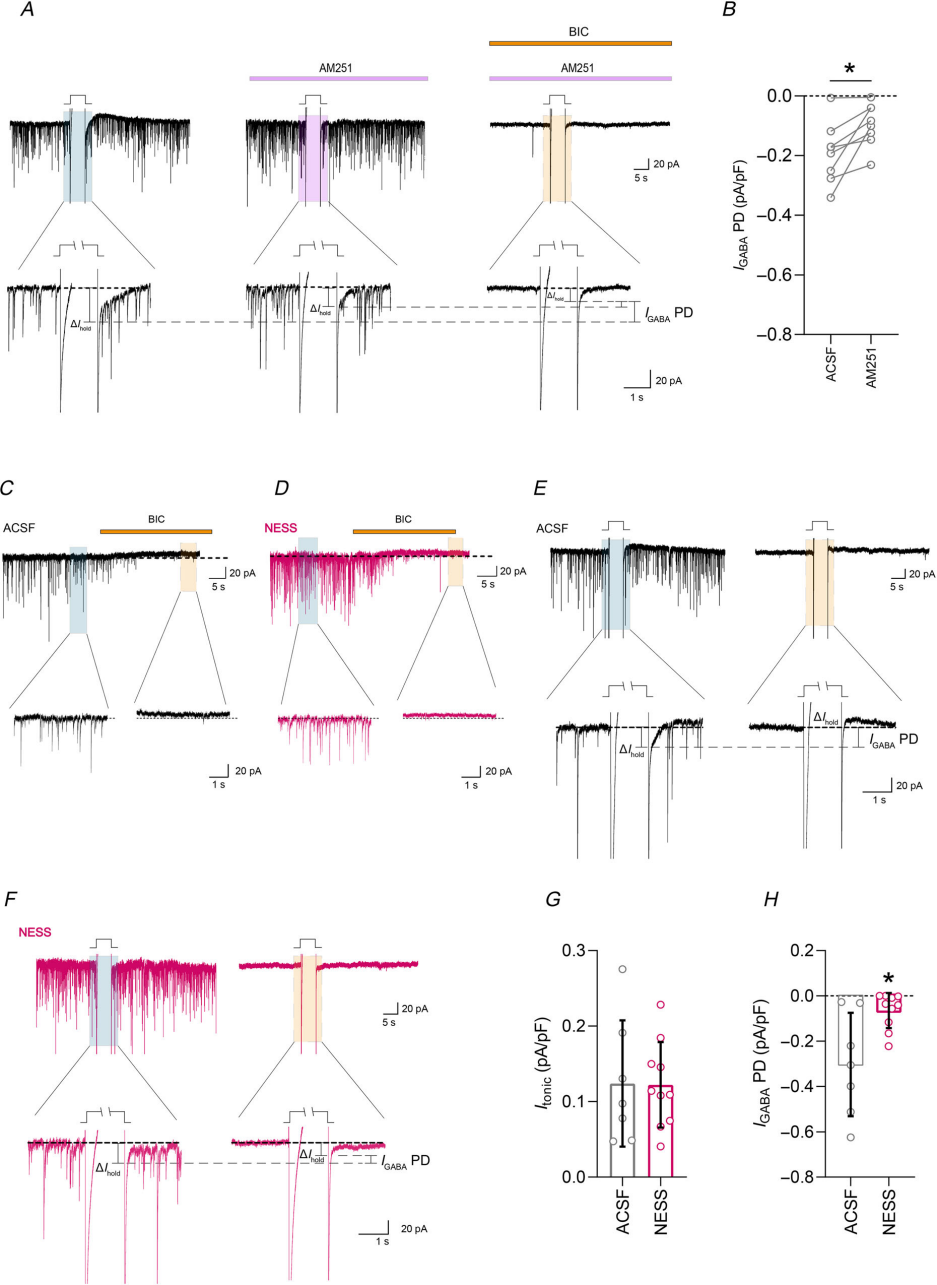
CB1 receptor contributes to eCB‐mediated potentiation of tonic GABA inhibition *A*, representative recordings illustrating the sequential protocol used to quantify the Δ*I*_hold_ and the corresponding BIC‑sensitive component of Δ*I*_hold_ (*I*_GABA_ PD) in a within‐cell approach. Three consecutive conditions were applied to the same neuron: ACSF, AM251 (10 µM), and BIC (20 µM) co‐applied with AM251 (as indicated by the overlapping orange and pink drug bars). Below each full trace, expanded segments corresponding to the ACSF (cyan box), AM251 (pink box) and BIC + AM251 (orange box) epochs are shown. All three segments are from the same continuously recorded neuron. The depolarising step is truncated, with only its onset and offset visible. Dashed lines are shared across all three insets to allow direct visual comparison of Δ*I*_hold_ magnitude; they indicate the baseline *I*_hold_ and the peak Δ*I*_hold_ level. *I*_GABA_ PD denotes the BIC‐sensitive component used to compute the depolarisation‐induced tonic current after normalisation. *B*, summary plot showing the effect of CB1 receptor blockade on the depolarisation‑evoked GABA current (Δ*I*_GABA_ PD) in within‑cell paired recordings. Individual cells are connected by lines to highlight the within‑cell comparison, and AM251 produced a significant reduction in Δ*I*_GABA_ PD (paired *t* test: *P = 0.015). *C* and *D*, representative current traces from cortical pyramidal neurons recorded in ACSF (*C*) and in the continuous presence of the neutral CB1 antagonist NESS‑0327 (NESS, 0.5 µM; *D*), before and during BIC application (orange bar). Below each full trace, 5 s segments corresponding to the cyan (pre‐BIC) and orange (post‐BIC) epochs are shown at an expanded timescale. Dashed lines indicate the holding current level in the pre‐BIC condition. *E* and *F*, representative continuous full‐trace recordings from ACSF‐ (*E*) and NESS‐ (*F*) treated neurons, obtained during ACSF (left) and BIC (right) superfusion, each showing the response to a depolarising step (truncated). Below each full trace, expanded segments corresponding to the cyan (pre‐BIC) and orange (post‐BIC) epochs illustrate 2 s of baseline activity followed by 2 s after the depolarising step. The depolarising step is truncated, with only its onset and offset visible (bracket symbols). Dashed lines are shared across pre‐BIC and post‐BIC insets to allow direct visual comparison of Δ*I*_hold_ magnitude; they indicate the baseline *I*_hold_ and the peak Δ*I*_hold_ level. *I*_GABA_ PD denotes the BIC‐sensitive component used to compute the depolarisation‐induced tonic current. *G*, summary data showing tonic BIC‑sensitive tonic current (*I*_tonic_) recorded in ACSF and NESS conditions. No significant difference was observed between the two groups, indicating NESS does not affect basal extrasynaptic GABAergic tone. *H*, summary data showing the depolarisation‑evoked GABA current (*I*_GABA_ PD) measured in ACSF and NESS‑0327 conditions, revealing a significantly reduced *I*_GABA_ PD in NESS‐treated slices compared to ACSF (*P = 0.032; Mann–Whitney test). Data are expressed as mean ± SD with each circle representing a single cell recorded.

Given that in our previous experiments AM251 did not significantly prevent *I*_GABA_ PD, despite the statistically significant suppression of *I*_GABA_ PD by the DAGL inhibitor DO34, we sought to better clarify the role of CB1 receptors by adopting a more sensitive within‑cell experimental design. To this end, we first recorded Δ*I*_hold_ following depolarisation under control conditions, and subsequently, in the same neuron, we repeated the depolarisation step 15 min after AM251 (10 µM) wash‑in. Finally, depolarisation was again repeated in the presence of BIC co‑applied with AM251 to isolate the GABA‑sensitive component of Δ*I*_hold_ (Fig. 4*A*). This within‑cell approach was chosen to minimise possible inter‑cell variability. Under these conditions, AM251 significantly attenuated, but not abolished, Δ*I*_GABA_ PD [*n*_(cells)_ = 8, *N*_(mice)_ = 5; paired *t* test: *t*_(7)_ = 3.130, *P* = 0.017; Fig. 4*B*], confirming that CB1 receptor activation contributes to eCB‑mediated potentiation of tonic GABA current and suggesting that the effect of AM251 in the between‐cell approach may be masked by inter‑cell variance.

To further validate these findings and to exclude potential confounding effects arising from the inverse‑agonist properties of AM251, we tested the effect of the neutral CB1 antagonist NESS‑0327(NESS; 0.5 µM) on *I*_tonic_ (Fig. 4*C*–*D*) and on *I*_GABA_ PD (Fig. 4*E*–*F*), in a separate set of between‑cell experiments. NESS had no effect on basal tonic current relative to the control condition [unpaired *t* test, *t*_(15)_ = 0.054, *P* = 0.958; Fig. 4*G*]. In contrast to the results obtained with AM251, NESS [*n*_(cells)_ = 10, *N*_(mice)_ = 3] significantly reduced *I*_GABA_ PD compared to vehicle‑treated cells [*n*_(cells)_ = 7, *N*_(mice)_ = 4; Mann–Whitney test, *P* = 0.032; Fig. 4*H*]. These results strengthen the notion that the depolarisation‑induced increase in tonic GABAergic transmission involves the activation of CB1 receptors.

### Boosting 2‐AG synthesis increases depolarisation‐induced potentiation of tonic GABA inhibition

**Figure 5:**
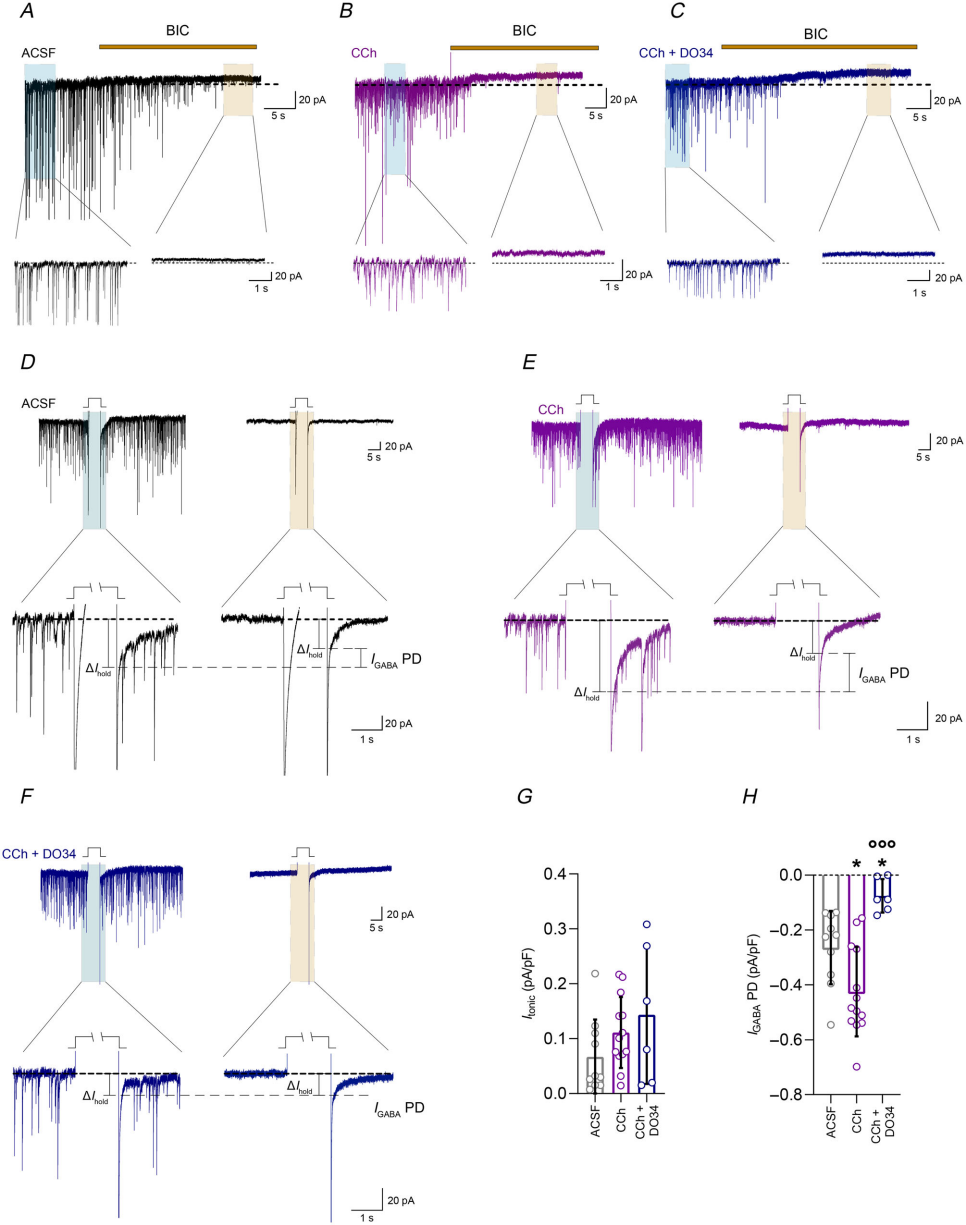
2‐AG synthesis augmentation enhances depolarisation‐induced potentiation of tonic GABA inhibition *A*–*C*, representative current traces recorded from cortical pyramidal neurons in ACSF (*A*), in the continuous presence of the muscarinic receptor agonist carbachol (CCh, 5 µM; *B*), and in slices continuously superfused with CCh and pre‐incubated with DO34 (CCh + DO34; *C*). BIC application (20 µM; orange bar) abolished spontaneous IPSCs and produced a comparable outward shift in the holding current (*I*_tonic_) across all conditions. Below each full‐trace, 5 s segments corresponding to the cyan (pre‐BIC) and orange (post‐BIC) epochs are shown at an expanded timescale. Dashed lines in each inset indicate the baseline (pre‐BIC) holding current level. *D*–*F*, representative continuous full‐trace recordings from ACSF‐ (*D*), CCh‐ (*E*) and CCh + DO34‐ (*F*) treated slices obtained during pre‐BIC (left) and BIC (right) superfusion, each showing the response to a depolarising step (truncated). Below each full‐trace, expanded segments corresponding to the cyan (pre‐BIC) and orange (BIC) epochs illustrate 2 s of baseline activity followed by 2 s after the depolarising step. The depolarising step is truncated, with only its onset and offset visible (bracket symbols). Dashed lines are shared between the left and right insets to allow direct visual comparison of Δ*I*_hold_ magnitude; they indicate the baseline *I*_hold_ and the peak Δ*I*_hold_ level. *I*_GABA_ PD denotes the BIC‐sensitive component used to compute the depolarisation‐induced tonic current. *G*, summary data showing the magnitude of the BIC‑sensitive tonic current (*I*_tonic_) in ACSF, CCh and CCh + DO34 conditions. No significant differences were observed across conditions. *H*, summary data showing *I*_GABA_ PD in ACSF, CCh and CCh + DO34 conditions. CCh significantly increased *I*_GABA_ PD relative to ACSF (*P = 0.027 CCh *vs*. ACSF; Tukey's multiple comparisons test) whereas co‑application of DO34 with CCh prevented this potentiation (Tukey's multiple comparisons test; *P = 0.036 CCh + DO34 *vs*. ACSF; °°°*P* < 0.001 CCh + DO *vs*. CCh). Data are expressed as mean ± SD, with each circle representing a single cell recorded.

Our data indicated that the potentiation of the tonic GABA inhibition following neuronal depolarisation arises from the mobilisation of eCBs and binding to the CB1 receptor. Accordingly, we reasoned that boosting eCB synthesis should further potentiate the increased GABA tone following depolarisation. To test this possibility, we washed the slices with CCh, a muscarinic receptor agonist carbachol, that stimulates eCB signalling, particularly 2‐AG synthesis, in a Ca^2+^‐independent manner (Colangeli et al., 2023; Makara et al., 2007; Tanimura et al., 2010). We then tested basal *I*_tonic_ (Fig. 5*A*–*C*) and *I*_GABA_ PD (Fig. 5*D*–*F*). CCh [*n*_(cells)_ = 13; *N*_(mice)_ = 9] had no significant effect on *I*_tonic_ when compared with ACSF [*n*_(cells)_ = 10; *N*_(mice)_ = 4; Kruskal‐Wallis test; *H* = 2.469; *P* = 0.291; Fig. 5*G*], further suggesting that there is no basal eCB tone modulating tonic GABAergic inhibition in SI. Conversely, one‐way ANOVA (*F*_2,26_ = 13.50; *P* < 0.001) revealed a significant effect of CCh on depolarisation‐induced potentiation of tonic GABA inhibition. In particular, CCh [*n*_(cells)_ = 13; *N*_(mice)_ = 9] significantly potentiated *I*_GABA_ PD with respect to the ACSF condition [*n*_(cells)_ = 10; *N*_(mice)_ = 4; Tukey's multiple comparisons test; *P* = 0.027 ACSF *vs*. CCh; Fig. 5*H*]. These data corroborate the hypothesis that eCB signalling drives post‐depolarisation potentiation of tonic GABAergic inhibition. To rule out any effect of CCh not related to eCB signalling, we tested CCh in the presence of DO34. While preincubation of DO34 in combination with CCh [*n*_(cells)_ = 6; *N*_(mice)_ = 4] had no effect on *I*_tonic_ (Fig. 5*G*), the DO34 + CCh combination did significantly reduce *I*_GABA_ PD when compared to ACSF (Tukey's multiple comparisons test; *P = 0.036) and CCh alone (Tukey's multiple comparisons test; ^ooo^ *P* < 0.001; Fig. 5*H*). These observations provide evidence that eCB mobilisation, particularly 2‐AG mobilisation, is required for tuning tonic GABAergic inhibition following depolarisation.

### Enhancing ambient GABA by inhibiting its reuptake does not occlude depolarisation‐induced potentiation of tonic GABA inhibition

**Figure 6:**
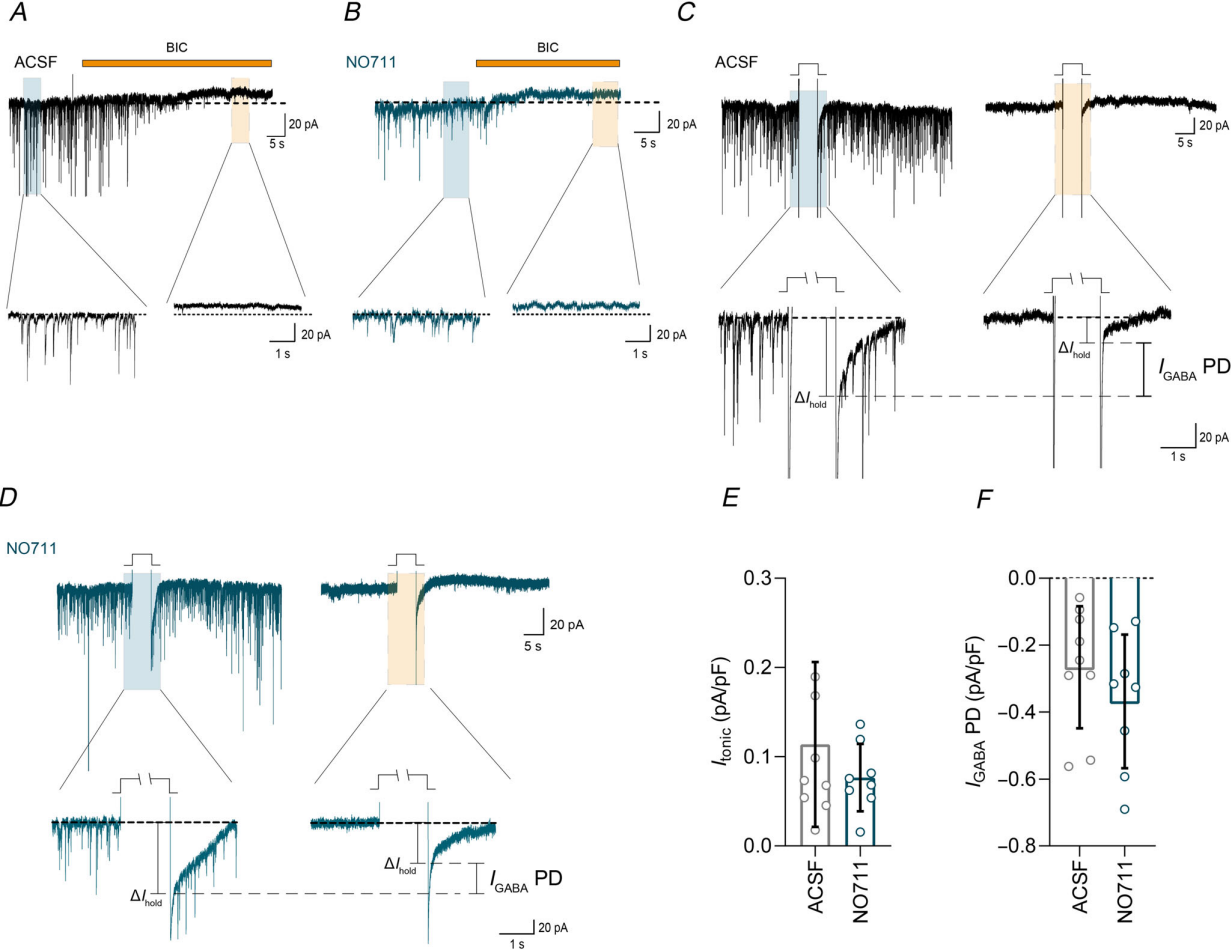
Depolarisation‐induced potentiation of tonic GABA inhibition is insensitive to inhibition of GAT‐1 *A* and *B*, representative full‐trace recordings from cortical pyramidal neurons in ACSF (*A*) and in the continuous presence of the GAT‐1 blocker NO711 (20 µM; *B*), under control conditions and during BIC application (20 µM; orange bar). BIC abolished spontaneous IPSCs and produced an outward shift in the holding current in both conditions. Below each full‐trace, 5 s segments corresponding to the cyan (pre‐BIC) and orange (post‐BIC) epochs are shown at an expanded timescale. Dashed lines in each inset indicate the drug‐free baseline holding current level to facilitate visual comparison. *C* and *D*, representative continuous full‑trace recordings from ACSF‐ (*C*) and NO711‐ (*D*) treated slices obtained during pre‐BIC (left) and BIC (right) superfusion, each showing the response to a depolarising step (truncated). Below each full‐trace, expanded segments corresponding to the cyan (pre‐BIC) and orange (BIC) epochs illustrate 2 s of baseline activity followed by 2 s after the depolarising step. The depolarising step is truncated, with only its onset and offset visible (bracket symbols). Dashed lines are shared between the left and right insets to allow direct visual comparison of Δ*I*_hold_ magnitude; they indicate the baseline *I*_hold_ and the peak Δ*I*_hold_ level. *I*_GABA_ PD denotes the BIC‐sensitive component used to compute the depolarisation‐induced tonic current. *E*, summary data showing the magnitude of the BIC‑sensitive tonic current (*I*_tonic_) in ACSF and NO711 conditions. No significant differences were observed between groups. *F*, summary data showing *I*_GABA_ PD in ACSF and NO711 conditions. Pretreatment with NO711 did not alter the depolarisation‑evoked increase in tonic GABA inhibition. Data are expressed as mean ± SD, with each circle representing a single recorded cell.

We then investigated the mechanism by which depolarisation‐induced eCB mobilisation promotes the transient augmentation of extrasynaptic GABA tone. Since depolarisation of postsynaptic neurons resulted in an eCB‐mediated suppression of presynaptic GABA release, it is reasonable to expect that it would also reduce tonic GABA activity. One hypothesis is that eCB signalling may have a dual action on GABA transmission: dampening synaptic GABA release from presynaptic terminals and increasing ambient GABA levels originating from neuronal or extraneuronal sources. To assess whether an elevation of extrasynaptic GABA concentration underlies the depolarisation‐induced potentiation of extrasynaptic GABA currents, we preincubated slices with the GAT‐1 inhibitor NO711 and tested *I*_tonic_ (Fig. 6*A*–*B*) and *I*_GABA_ PD (Fig. 6*C*–*D*). Indeed, if a depolarisation‐induced increase in GABAergic tone occurs through a transient increase in ambient GABA concentration, then blocking GABA uptake through GAT‑1 inhibition should occlude the depolarisation‐induced increase in tonic inhibition. We employed a GAT‐1 inhibitor to mimic increased ambient GABA concentration since GAT‐1 is widely expressed in SI (Conti et al., 2004; Fattorini et al., 2020; Melone et al., 2015; Minelli et al., 1995), and contributes to tonic GABAergic inhibition (Bragina et al., 2008). However, we found that, when compared to the ACSF condition [*n*_(cells)_ = 9; *N*_(mice)_ = 6], neither basal tonic inhibition (unpaired *t* test; *t*_15_ = 1.061.; *P* = 0.305; Fig. 6*E*] nor post‐depolarisation tonic current (unpaired *t* test; *t*_15_ = 1.098; *P* = 0.290; Fig. 6*F*) were affected by pretreatment with NO711 [*n*_(cells)_ = 8; *N*_(mice)_ = 3]. This observation suggests that eCB‐mediated potentiation of tonic GABA inhibition does not arise from a transient increase in ambient GABA concentration or from interaction with GAT‐1.

### GABA supplementation increases depolarisation‐induced potentiation of tonic GABA inhibition

Since GAT‐1 is not the only transporter responsible for the reuptake of GABA in the brain, as GAT‐3 also contributes to limiting GABA concentration in the extracellular space (Minelli et al., 1996) and because pre‐incubation of GAT‐1 inhibition might desensitise extrasynaptic GABA_A_ receptors, we applied GABA directly to the slice to quickly raise the ambient GABA concentration and verify whether it occludes post‐depolarisation potentiation of GABA tone.

**Figure 7:**
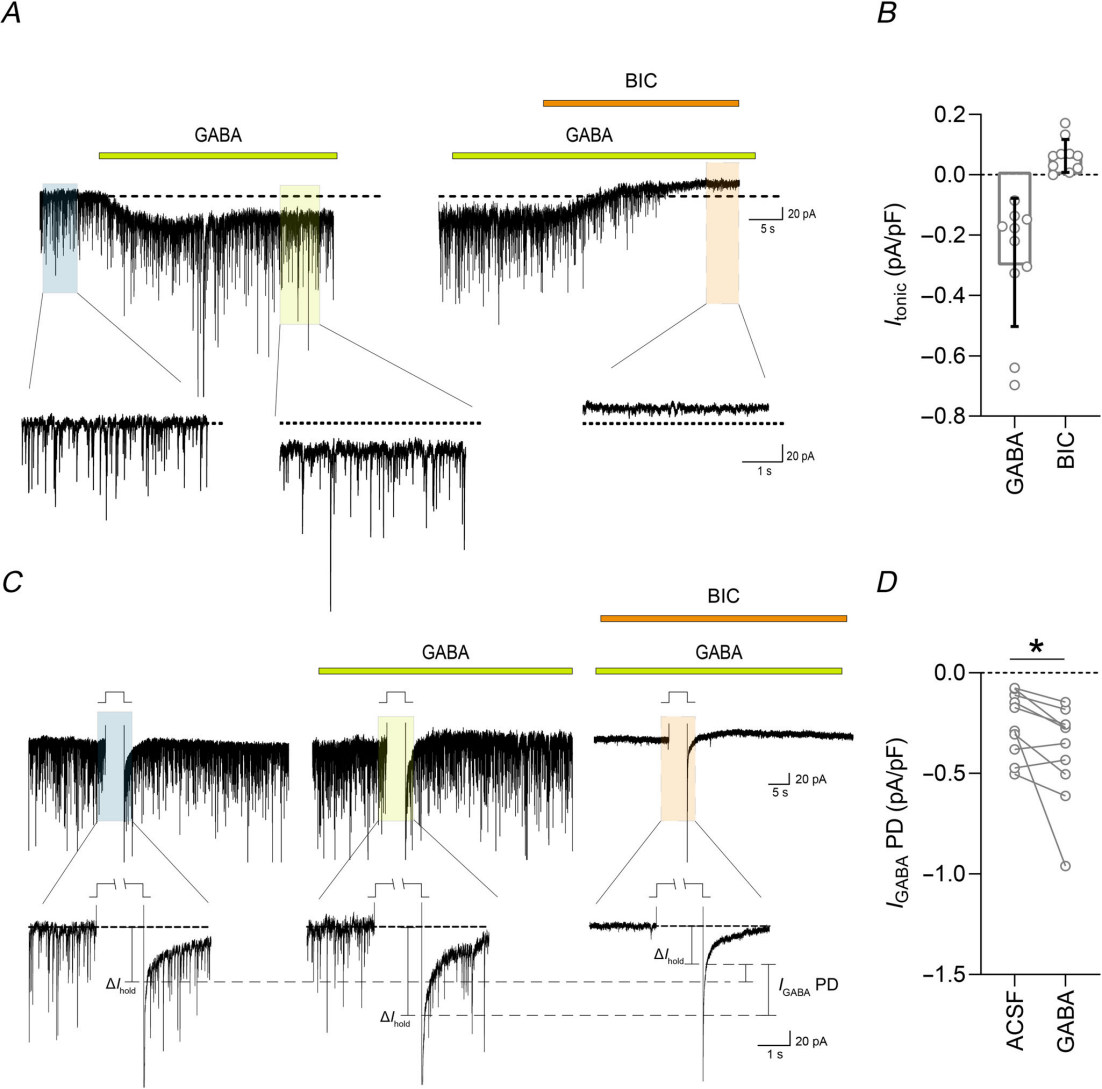
Depolarisation‐induced potentiation of tonic GABA inhibition is augmented rather than occluded when GABA is added in the ACSF *A*, representative current traces from cortical pyramidal neurons before and during the application of 2 µM GABA (green bar; left) and subsequent blockade of GABA_A_ receptors with 20 µM BIC during continuous GABA perfusion (as indicated by the overlapping orange and green drug bars, right). Colour‐coded boxes (cyan: drug‐free; green: GABA; orange: GABA + BIC) indicate the recording epochs shown at expanded timescale in the insets below. Each inset shows a 5 s segment; dashed lines in each inset indicate the drug‐free baseline *I*_hold_ level to facilitate visual comparison. *B*, graph showing quantification of the *I*_tonic_ calculated as the downward shift of the *I*_hold_ caused by GABA application and the subsequent outward shift provoked by BIC application with respect to the baseline drug‐free *I*_hold_. *C*, representative recordings illustrating the sequential protocol used to quantify Δ*I*_hold_ and the corresponding BIC‑sensitive component of Δ*I*_hold_ (*I*_GABA_ PD) in the within‑cell experiment. Three consecutive conditions were applied to the same neuron: ACSF, GABA (2 µM), and BIC (20 µM) co‐applied with GABA (as indicated by the overlapping orange and green drug bars). Below each full trace, expanded segments corresponding to the ACSF (cyan box), GABA (green box) and BIC + GABA (orange box) epochs are shown. All three segments are from the same recorded neuron. The depolarising step is truncated, with only its onset and offset visible. Dashed lines are shared across all three insets to allow direct visual comparison of Δ*I*_hold_ magnitude; they indicate the baseline *I*_hold_ and the peak Δ*I*_hold_ level. *I*_GABA_ PD denotes the BIC‐sensitive component used to compute the depolarisation‐induced tonic current. *D*, summary plot showing that GABA significantly potentiated the depolarisation‑evoked tonic current (Δ*I*_GABA_ PD) in within‑cell paired recordings (*P = 0.048, paired *t* test). Individual cells are connected by lines to highlight the within‑cell comparison. Data are expressed as mean ± SD, with each circle representing a single cell recorded.

We first assessed whether exogenously applied GABA increases *I*_tonic_ without desensitising extrasynaptic GABA_A_ receptors (Fig. 7*A*). At a concentration of 2 µM, GABA application produced a robust increase in the tonic current, revealed by a downward shift of *I*_hold_ by −19.69 ± 10.01 pA [*n*_(cells)_ = 10; *N*_(mice)_ = 5, Fig. 7*B*]. The addition of BIC caused a large upward shift that overpassed the initial *I*_hold_ by 4.91 ± 4.57 pA (Fig. 7*B*). This suggested that exogenously applied GABA potentiates tonic inhibition without causing desensitisation of extrasynaptic GABA_A_ receptors. We next verified whether increased ambient GABA may underlie the post‐depolarisation potentiation of GABAergic tonic current; in this case, exogenously applied GABA should occlude the post‐depolarisation potentiation of tonic GABA current. To this end, we performed a within‐cell paradigm by applying a depolarisation step while superfusing ACSF only and, in the same neuron, repeating the depolarisation step after GABA wash‐in. We then measured Δ*I*_hold_ before and after GABA application and, ultimately, we measured Δ*I*_hold_ after BIC application to isolate *I*_GABA_ PD (Fig. 7*C*). When we compared *I*_GABA_ PD in ACSF and GABA conditions we found that enhancing extracellular GABA concentration further potentiated rather than occluded *I*_GABA_PD (*P* = 0.048, paired *t* test; Fig. 7*D*). This finding rules out the possibility that the potentiation of GABA tone arises from a transient increase in extrasynaptic GABA concentration and indicates that the lack of effect observed with NO711 cannot be attributed to a ceiling effect, as elevating extracellular GABA enhanced rather than saturated the plasticity of tonic inhibition.

### Inhibition of neurosteroid biosynthesis prevents depolarisation‐induced increase of tonic GABA inhibition

**Figure 8:**
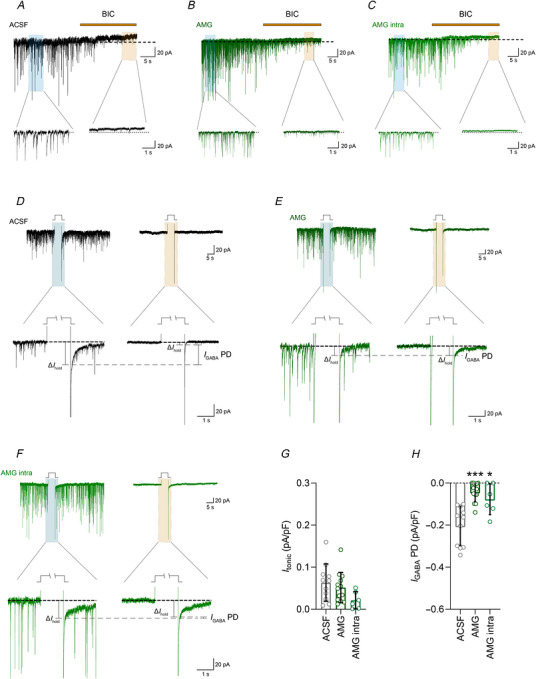
Inhibition of neurosteroid synthesis cascade prevents depolarisation‐induced potentiation of tonic GABA inhibition *A*–*C*, representative current traces recorded from cortical pyramidal neurons in ACSF (*A*), during bath application of AMG (*B*) and with AMG‐intra delivered through the recording pipette (*C*). BIC application (20 µM; orange bar) abolished spontaneous IPSCs and produced a comparable outward shift in the holding current (*I*_tonic_) across all conditions. Below each full‐trace, 5 s segments corresponding to the cyan (pre‐BIC) and orange (post‐BIC) epochs are shown at an expanded timescale. Dashed lines in each inset indicate the baseline (pre‐BIC) holding current level. *D*–*F*, representative continuous full‐trace recordings from ACSF‐ (*D*), AMG‐ (*E*) and AMG‑intra‐ (*F*) treated neurons obtained during pre‐BIC (left) and BIC (right) superfusion, each showing the response to a depolarising step (truncated). Below each full‐trace, expanded segments corresponding to the cyan (pre‐BIC) and orange (BIC) epochs illustrate 2 s of baseline activity followed by 2 s after the depolarising step. The depolarising step is truncated, with only its onset and offset visible (bracket symbols). Dashed lines are shared between the left and right insets to allow direct visual comparison of Δ*I*_hold_ magnitude; they indicate the baseline *I*_hold_ and the peak Δ*I*_hold_ level. *I*_GABA_ PD denotes the BIC‐sensitive component used to compute the depolarisation‐induced tonic current. *G*, summary data showing the magnitude of the BIC‑sensitive basal tonic current (*I*_tonic_) in ACSF, AMG and AMG‑intra conditions. No significant differences were observed across groups. *H*, summary data showing *I*_GABA_ PD in ACSF, AMG and AMG‑intra conditions. AMG significantly reduced the depolarisation‑induced increase in tonic GABA inhibition, both when bath‑applied (***P < 0.001; ACSF *vs*. AMG; Dunn's multiple comparisons test) and when delivered intracellularly (*P = 0.039; ACSF *vs*. AMG‐intra; Dunn's multiple comparisons test). Data are expressed as mean ± SD, with each circle representing a single recorded cell.

Our findings showed that the potentiated extrasynaptic GABA currents observed following depolarisation require eCB signalling and are not mediated by transient changes in ambient GABA concentration. Moreover, the observation that increased GABA concentration also potentiated depolarisation‐induced augmentation of GABA tone raised the possibility that a neuromediator released upon depolarisation may synergistically act at extrasynaptic GABA_A_ receptors to contribute to tuning GABAergic tone under sustained neuronal activity. Neurosteroids affect both synaptic and extrasynaptic GABAergic inhibition through potentiation of GABA_A_ receptors at low concentrations (Carver & Reddy, 2013), produce changes in neuronal excitability on a short timescale (Carver & Reddy, 2013), and their synthesis can be stimulated by cannabinoids (Raux & Vallée, 2023). This prompted us to investigate whether locally synthesised neurosteroids contribute to this form of tonic GABAergic plasticity. We used AMG, an inhibitor of the mitochondrial cytochrome P450scc, the enzyme required for the biosynthesis of all neurosteroids (Tanaka & Sokabe, 2012). We therefore investigated the effect of AMG on basal tonic GABAergic inhibition under basal condition (Fig. 8*A*–*C*) and under sustained neuronal activity (Fig. 8*D*–*F*). Since neurosteroids can be produced in both neuronal and non‐neuronal cells, we decided to administer AMG either in the slice to broadly inhibit neurosteroid production or in the recorded cell only through the recording pipette to assess a putative autocrine action of neurosteroids. We first assessed whether there was an endogenous neurosteroid tone able to tune basal tonic GABAergic inhibition. In this case AMG should reveal this effect by dampening *I*_tonic_. We recorded *I*_tonic_ in the presence of ACSF only [*n*_(cells)_ = 11; *N*_(mice)_ = 5; Fig. 8*A*], AMG superfused on the slice [*n*_(cells)_ = 13; *N*_(mice)_ = 6; Fig. 8*B*] or the AMG delivered through the recording pipette [*n*_(cells)_ = 6; *N*_(mice)_ = 2; Fig. 8*C*]. We found no statistical difference between groups, indicating that there is no endogenous neurosteroid tone affecting basal tonic GABAergic inhibition (Kruskal–Wallis test; *H* = 4.969, *P* = 0.083; Fig. 8*G*). We then examined whether stimulus‐dependent changes in tonic GABAergic inhibition depended on neurosteroid action. We tested *I*_GABA_ PD in the presence of AMG either bath applied [*n*_(cells)_ = 13; *N*_(mice)_ = 6; Fig. 8*E*] or delivered through the recording pipette [*n*_(cells)_ = 6; *N*_(mice)_ = 2; Fig. 8*F*], and found a significant effect of AMG in blunting depolarisation‐induced enhanced GABA tone (Kruskal–Wallis test; *H* = 16.00, *P* < 0.001; Fig. 8*H*). Dunn's multiple comparisons test revealed that AMG significantly reduced tonic GABA plasticity either when bath applied (***P < 0.001; ACSF *vs*. AMG) or delivered through the recording pipette (*P = 0.039; ACSF *vs*. AMG intra). This observation provides evidence that tonic GABA plasticity requires *de novo* neurosteroid synthesis.

### Gabazine confirms the eCB‑dependent GABAergic component of *I*_GABA_ PD without revealing basal tonic inhibition

BIC is widely used to quantify tonic GABA receptor‐mediated currents; however, in addition to blocking GABA receptors, BIC can influence spontaneous channel openings (Bai et al., 2001) and inhibit Ca^2+^‐activated K^+^ channels (Khawaled et al., 1999). Thus, the BIC‑sensitive component of *I*_GABA_ PD could, in principle, arise from Ca^2+^‐dependent K^+^ conductances rather than from genuine potentiation of extrasynaptic GABA_A_ receptors.

**Figure 9:**
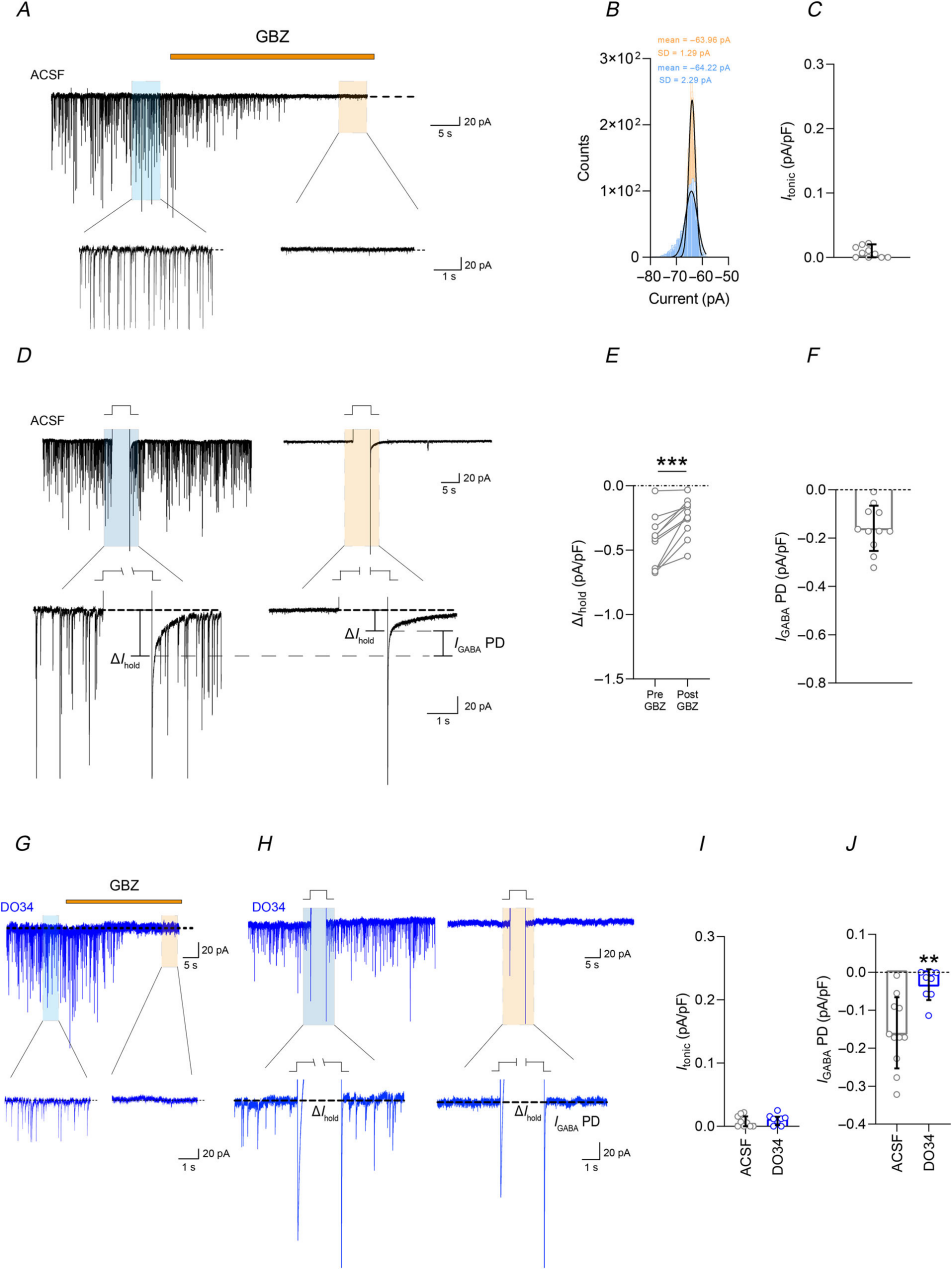
GBZ failed to reveal *I*_tonic_ while confirming the GABAergic component of the 2‐AG‐mediated *I*_GABA_ PD *A*, representative full‑trace recording of tonic and synaptic GABAergic currents in mouse cortical neurons. Bath application of the selective GABA_A_ receptor antagonist gabazine (GBZ, 100 µM; orange bar) abolished spontaneous IPSCs but failed to produce an outward shift in the holding current (*I*_tonic_). Below, 5 s segments of the same traces are shown at an expanded timescale, illustrating the holding current during control (cyan) and GBZ (orange) conditions. Dashed lines in each inset indicate the baseline (pre‐GBZ) *I*_hold_ level. *B*, current distributions (5 s epochs sampled in 10 ms segments every 100 ms) were fitted with Gaussian functions to extract the mean holding current before (cyan) and after GBZ (orange) application. Embedded text shows the mean ± SD of each distribution. GBZ provoked no shift of the Gaussian peak from the recording shown in *A*. *C*, bar graph showing GBZ‐sensitive *I*_tonic_ normalised to cell capacitance (pA/pF). *D*, representative continuous full‑trace recordings obtained before and after the depolarisation step (truncated) during ACSF (pre‐GBZ; left) and GBZ superfusion (right). Below, expanded timescale segments corresponding to the cyan (pre‐GBZ) and orange (post‐GBZ) epochs illustrate 2 s of baseline activity followed by 2 s after the depolarising step. The depolarising step is truncated, with only its onset and offset visible (bracket symbols). Dashed lines are shared between the left and right insets to allow direct visual comparison of Δ*I*_hold_ magnitude; they indicate the baseline *I*_hold_ and the peak Δ*I*_hold_ level. *I*_GABA_ PD denotes the GBZ‐sensitive component used to compute the depolarisation‐induced tonic current. *E*, paired graph showing Δ*I*_hold_ (pA/pF) before (Pre‐GBZ) and after (Post‐GBZ) GBZ application. The depolarisation‐induced downward shift of the holding current was significantly attenuated in the presence of GBZ (***P < 0.001; paired *t* test). *F*, bar graph showing the GBZ‐sensitive component of the depolarisation‐induced Δ*I*_hold_ (*I*_GABA_ PD), calculated and normalised to cell capacitance (pA/pF). *G*, representative traces showing that in the presence of DO34, GBZ failed to induce a shift in *I*_hold._ Below, 5 s segments of the same traces are shown at an expanded timescale, illustrating *I*_hold_ during control (cyan) and GBZ (orange) conditions in DO34‐treated slices. *H*, representative continuous full‑trace recordings obtained before and after the depolarisation step (truncated) during pre‑BIC (left) and post‑BIC superfusion (right) from DO34‐treated slices. Below, expanded timescale segments corresponding to the cyan (pre‐GBZ) and orange (post‐GBZ) epochs illustrate 2 s of baseline activity followed by 2 s after the depolarising step. The depolarising step is truncated, with only its onset and offset visible (bracket symbols). Dashed lines are shared between the left and right insets to allow direct visual comparison of Δ*I*_hold_ magnitude; they indicate the baseline *I*_hold_ and the peak Δ*I*_hold_ level. *I*_GABA_ PD denotes the GBZ‐sensitive component used to compute the depolarisation‐induced tonic current. *I*, summary data showing the magnitude of the basal tonic GBZ‑sensitive current (*I*_tonic_) in DO34‐treated slices compared to ACSF control condition. No significant differences were observed across groups. *J*, summary data showing GBZ‐sensitive *I*_GABA_ PD in DO34‐treated slices compared to ACSF‐treated conditions. DO34 significantly reduced the depolarisation‑induced increase in tonic GABA inhibition (**P = 0.004; Mann‐Whitney test). Data are expressed as mean ± SD, with each circle representing an individual recorded cell.

To address this issue, we repeated the experiments using GBZ (SR‑95531), a more selective competitive GABA_A_ antagonist that does not affect Ca^2+^‐activated K^+^ channels (Ueno et al., 1997). Low concentrations of GBZ preferentially block synaptic GABA receptors, whereas higher concentrations can also reveal tonic GABA currents (Stell & Mody, 2002). We then tested whether a saturating dose of 100 µM GBZ (Cope et al., 2009) unmasks a tonic GABAergic current in cortical pyramidal neurons (Fig. 9*A*). Surprisingly, we found that GBZ failed to reveal a basal tonic current in our preparation [*I*_tonic_ = 0.6945 ± 1.823 pA; Wilcoxon test: *P* = 0.297; *n*_(cells)_ _=_ 11, *N*_(mice)_ = 5; Fig. 9*B*–*C*]. This finding indicates that the BIC‑sensitive basal tonic current in somatic sensory cortex may arise either from spontaneously active extrasynaptic receptors or from BIC‑sensitive K^+^ conductances, and that basal ambient GABA concentration is probably not sufficient to tonically activate extrasynaptic GABA_A_ receptors. This unexpected result prompted us to verify whether depolarisation‐induced ΔI_hold_ depends on extrasynaptic GABA_A_ receptor activity. We therefore tested Δ*I*_hold_ before and after GBZ application (Fig. 9*D*), and found that GBZ significantly attenuated the depolarisation‑evoked Δ*I*_hold_ [paired *t* test; *t*_(10)_ = 5.336, *P* < 0.001; Fig. 9*E*]. The extrapolated GBZ‐sensitive component of Δ*I*_hold_ [−16.50 ± 11.51 pA; *n*_(cells)_ = 11; *N*_(mice)_ = 5] normalised for *C*_m_ is shown in Fig. 9*F*. Thus, GBZ mirrored the effect of BIC, providing evidence that the post‑depolarisation shift in holding current is mediated by GABA_A_ receptor activity rather than by off‐target effects of BIC.

To confirm that the GBZ‑sensitive post‑depolarisation potentiation of tonic GABA current is gated by eCB signalling, we pre‐incubated slices with DO34 [*n*_(cells)_ _=_ 8, *N*_(mice)_ = 3] and then tested the effect of GBZ on *I*_tonic_ (Fig. 9*G*) and *I*_GABA_ PD (Fig. 9*H*). Again, we found that in the presence of DO34, GBZ was not able to reveal a tonic current (Mann–Whitney test; *P* = 0.293, ACSF *vs*. DO34; Fig. 9*I*). Furthermore, as observed in the BIC experiments, DO34 markedly reduced the GBZ‑sensitive component of the depolarisation‑induced current with respect to ACSF condition (Mann–Whitney test; *P* = 0.004; Fig. 9*J*), indicating that the GBZ‐sensitive *I*_GABA_ PD requires on‑demand 2‑AG mobilisation. Together, these findings confirm that the depolarisation‑induced potentiation of tonic inhibition is mediated by eCB‑dependent enhancement of extrasynaptic GABAergic receptor activity.

### The neurosteroid ALLO contributes to extrasynaptic GABA_A_ receptor activation

**Figure 10:**
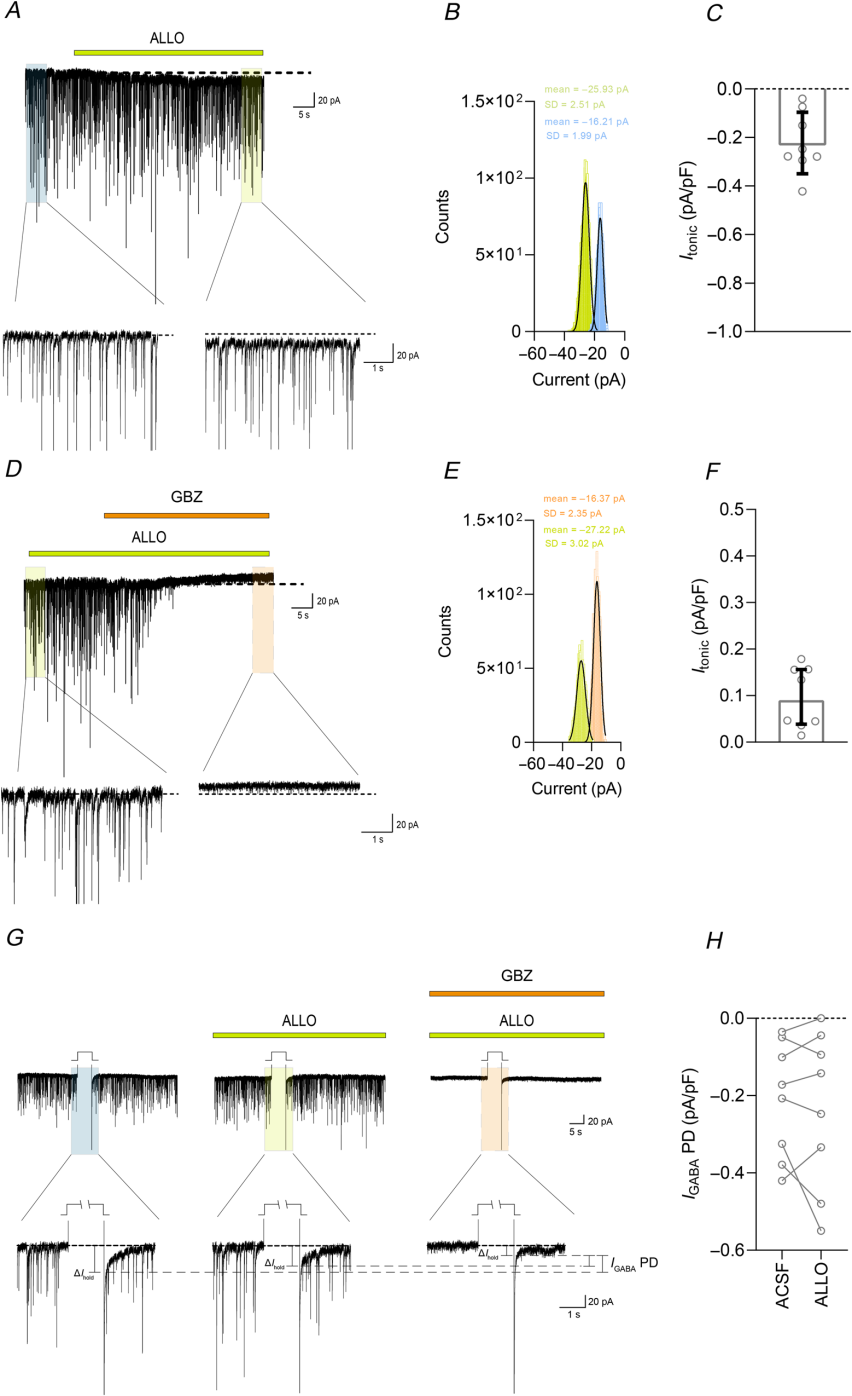
ALLO unmasks GBZ‐sensitive *I*_tonic_ and occludes GBZ‐sensitive *I*_GABA_ PD *A*, representative current traces from cortical pyramidal neurons recorded before and during application of the neurosteroid allopregnanolone (ALLO 1 µM) (green bar). Below, 5 s segments of the same traces are shown at an expanded timescale, illustrating the holding current during control (cyan) and ALLO (green) conditions. Dashed lines in each inset indicate the baseline (pre‐ALLO) holding current level. *B*, current distributions (5 s epochs sampled in 10 ms segments every 100 ms) were fitted with Gaussian functions to extract the mean holding current before (cyan) and after ALLO (green) application (*I*_tonic_). Embedded text shows the mean ± SD of each distribution. The leftward shift in the Gaussian peak indicates the inward *I*_tonic_ produced by ALLO refering to the trace shown in *A*. *C*, bar graph showing the inward *I*_tonic_ produced by ALLO normalised to cell capacitance (pA/pF). *D*, representative current traces from cortical pyramidal neurons recorded in the continuos presence of ALLO (1 µM; green bar) and after the subsequent co‐application of GBZ (100 µM; orange bar). Below, 5 s segments of the same traces are shown at an expanded timescale, illustrating *I*_hold_ during ALLO (green) and ALLO + GBZ (green and orange) conditions. Dashed lines in each inset indicate the holding current level before GBZ application and during ALLO superfusion. *E*, current distributions (5 s epochs sampled in 10 ms sweeps every 100 ms) were fitted with Gaussian functions to extract the mean *I*_hold_ before and after GBZ application during continuous ALLO superfusion. The rightward shift in the Gaussian peak indicates that, in the presence of ALLO, GBZ did reveal an *I*_tonic_ in the trace shown in *A*. *F*, bar graph showing the outward *I*_tonic_ provoked by GBZ during continuous ALLO superfusion normalised to cell capacitance (pA/pF). *G*, representative within‑cell recording illustrating the sequential protocol used to quantify Δ*I*_hold_ and the corresponding GBZ‑sensitive component of Δ*I*_hold_ (*I*_GABA_ PD). Three consecutive conditions were applied to the same neuron: ACSF, ALLO (1 µM), and GBZ (100 µM) co‐applied with ALLO (as indicated by the overlapping orange and green drug bars). Below each full trace, expanded segments corresponding to the ACSF (cyan box), ALLO (green box) and GBZ + ALLO (orange box) epochs are shown. All three segments are from the same continuously recorded neuron. The depolarising step is truncated, with only its onset and offset visible. Dashed lines are shared across all three insets to allow direct visual comparison of Δ*I*_hold_ magnitude; they indicate the baseline *I*_hold_ and the peak Δ*I*_hold_ level. *I*_GABA_ PD denotes the GBZ‐sensitive component used to compute the depolarisation‐induced tonic current. *H*, summary plot showing the effect of ALLO on the depolarisation‑evoked tonic current (Δ*I*_GABA_ PD) in within‑cell paired recordings. Individual cells are connected by lines to highlight the within‑cell comparison. ALLO did not produce a significant potentiation in Δ*I*_GABA_ PD. Data are expressed as mean ± SD, with each circle representing a single cell recorded.

We showed that under basal conditions ambient GABA is not sufficient to tonically activate extrasynaptic GABA receptors. In contrast, after depolarisation, these receptors become transiently engaged, generating a tonic current that depends on both eCB production and neurosteroid synthesis. In this scenario, depolarisation‑induced neurosteroid production may sensitise extrasynaptic GABA receptors, enabling the shift in *I*_hold_. If this is the case, exogenously raising neurosteroid levels should be sufficient to sensitise extrasynaptic GABA receptors and, by mimicking the depolarisation‑induced shift, produce per se an inward tonic current. We therefore tested the effect of ALLO on basal *I*_hold_ (Fig. 10*A*–*B*). We chose ALLO because, among the various neurosteroids generated by P450scc activity, it exerts potent modulatory actions on GABA receptors, with a particularly strong efficacy at δ‑subunit‑containing extrasynaptic GABA_A_ receptors (Belelli & Lambert, 2005). Consistent with this hypothesis, we found that ALLO [*n*_(cells)_ = 8, *N*_(mice)_ = 6] was able to provoke a downward shift in basal *I*_hold_ (−13.35 ± 5.68 pA), which was statistically significant [one‐sample *t* test: *t*_(7)_ _=_ 4.982, *P* = 0.0016; Fig. 10*C*]. This provides evidence that basal tonic conductance in somatic sensory cortex is sensitive to ALLO. Moreover, since the depolarisation‑evoked Δ*I*_hold_ is GBZ‑sensitive, ALLO application should render extrasynaptic GABA receptors responsive to GBZ even under basal conditions, thereby revealing a GBZ‑sensitive tonic current. Therefore, we examined the effect of GBZ application on tonic current while continuously superfusing slices with ALLO (Fig. 10*D*–*E*). We found that GBZ, which previously failed to reveal any basal tonic current, now unmasked a statistically significant [one‐sample *t* test; *t*_(7)_ _=_ 4.088, *P* = 0.0046; Fig. 10*F*] tonic conductance while ALLO was in the bath (6.251 ± 4.087 pA). This result provides direct evidence that neurosteroid‑dependent modulation of extrasynaptic GABA receptors is sufficient to generate a detectable GABAergic tonic current. The next step was to test the effect of ALLO directly on the GBZ‐sensitive *I*_GABA_ PD and determine whether it could further potentiate depolarisation‐induced potentiation of tonic inhibition. To this end, we performed within‑cell experiments testing depolarisation‐induced Δ*I*_hold_ in control conditions, after ALLO superfusion, and finally after GBZ application (Fig. 10*G*). ALLO failed to enhance *I*_GABA_ PD compared to the ACSF condition [paired *t* test; *t*_(7)_ _=_ 0.705, *P* = 0.503; Fig. 10*H*]. These data suggest that either ALLO is not the candidate neurosteroid responsible for *I*_GABA_ PD or that the depolarisation‑induced plasticity already recruits the full neurosteroid‑sensitive component of tonic inhibition.

## Discussion

Our data unveiled a form of plasticity of tonic GABA inhibition in the mouse cerebral cortex that emerges following neuronal depolarisation and requires the mobilisation of eCB, presumably 2‐AG, and the activation of CB1 receptors. The primary function of the eCB signalling is classically associated with the suppression of neurotransmitter release through its presynaptically expressed CB1 receptors (Kano et al., 2009; Soltesz et al., 2015). Our data provide evidence that, in addition to the activity‐dependent suppression of synaptic GABA transmission, depolarisation‑evoked eCB mobilisation transiently enhances extrasynaptic GABA inhibition in a CB1‑dependent manner. Tonic GABA inhibition occurs through activation of high‐affinity, extrasynaptic GABA_A_ receptors by low ambient GABA concentrations (Farrant & Nusser, 2005; Mody & Pearce, 2004; Semyanov et al., 2003, 2004). In the neocortex, the nature of the tonic GABA inhibition has been attributed to different GABA_A_ receptor subtypes (Marchionni et al., 2007), including α5‐, α1‐ and δ‐subunits (Yamada et al., 2007). Here, we show that *de novo* neurosteroid synthesis is required for the induction of this depolarisation‑evoked tonic GABA plasticity. Neurosteroids are potent modulators of GABA_A_ receptors, and δ‐subunit containing GABA_A_ receptors are particularly sensitive to the actions of neuroactive steroids (Belelli et al., 2009). This suggests that the δ‐subunit‐containing GABA_A_ receptors may be the primary mediators of the eCB‑dependent potentiation of tonic inhibition. Consistent with this idea, ALLO, known to have a powerful effect on the δ‐subunit‐containing GABA_A_ receptors (Belelli & Lambert, 2005), mimicked the depolarisation‐induced potentiation of tonic inhibition when applied exogenously. In addition, GBZ, which failed to reveal a detectable tonic conductance under baseline conditions, revealed tonic current when neurosteroid signalling was enhanced with ALLO. This indicates that endogenous neurosteroid and GABA tone are normally insufficient to activate extrasynaptic GABA_A_ receptors, yet these receptors are highly responsive to neurosteroid modulation. In this framework, the observation that depolarisation produces a GBZ‑sensitive increase in tonic inhibition indicates that activity can generate an endogenous neurosteroid tone strong enough to engage extrasynaptic receptors. The absence of further enhancement of depolarisation‐induced tonic inhibition in the presence of ALLO is consistent with an occlusion of this neurosteroid‑dependent pathway. Although we do not claim that ALLO is the specific neurosteroid mediating this form of plasticity, this set of experiments provides a proof‑of‑principle demonstration that neurosteroid activity is per se sufficient to engage this plasticity, and rules out potential off‑target effects of AMG, as P450scc inhibition may have broader consequences beyond suppressing neurosteroid synthesis, including possible alterations in mitochondrial function, redox balance and cholesterol trafficking.

**Figure 11:**
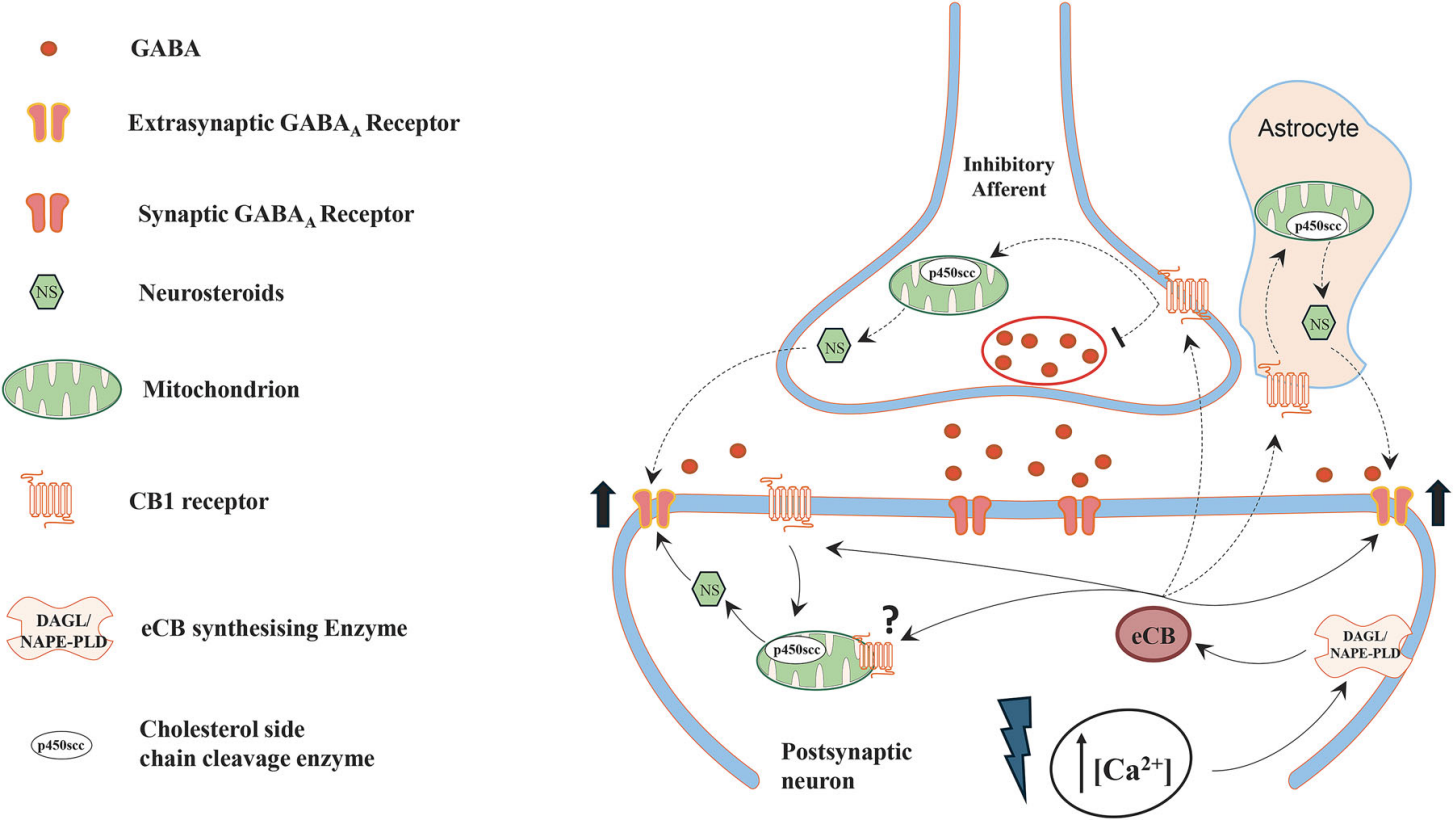
Possible mechanisms for eCB‐mediated potentiation of tonic GABA inhibition Depolarisation of postsynaptic neurons, allowing [Ca^2+^]_I_ elevations through voltage‐gated Ca^2+^ channels, triggers the production of eCB by eCB‐synthesising enzymes. eCBs may then act either as autocrine or paracrine mediators or both. In the mechanism proposed to underlie the autocrine action (solid arrows), eCBs remain in the postsynaptic membrane to bind to CB1 receptor expressed by the same cell. Somatodendritic (or mitochondrial) CB1 receptor activation then promotes *de novo* neurosteroidogenesis by probably enhancing the activity of the P450scc enzyme (Raux & Vallée, 2023) in the mitochondria of the postsynaptic neurons. Neurosteroids in turn can activate extrasynaptic GABA_A_ receptor without being released in the extracellular space. Moreover, eCBs may also potentiate tonic GABA inhibition in a CB1 receptor‐ and neurosteroid‐independent manner since there is evidence that 2‐AG is capable of directly binding to GABA_A_ receptors (Sigel et al., 2011). In the mechanism proposed to underlie the paracrine action (dashed arrows), once synthetised following [Ca^2+^]_I_ elevations, eCBs travel backwards across the synapse to bind CB1 receptor expressed in the presynaptic terminals or in the perisynaptic astrocyte processes. Besides the well‐known ability of presynaptic CB1 receptor to control neurotransmitter release, it can stimulate the production of neurosteroids in the mitochondria present in the axon terminals and astrocytes. Neurosteroids can be then released in the extracellular space to activate extrasynaptic GABA_A_ receptors and potentiate tonic GABA inhibition in the postsynaptic neuron.

Accumulating evidence indicates the existence of a bidirectional communication between the eCB system and neurosteroids (Vallée et al., 2014) including the ability of CB1 receptors to stimulate neurosteroid production (Raux & Vallée, 2023). It is therefore possible that following neuronal depolarisation, eCBs activate CB1 receptors to stimulate *de novo* neurosteroid synthesis to potentiate extrasynaptic GABA_A_ activity (Fig. 11). Besides its primary localisation in presynaptic terminals and astrocytes (Colangeli et al., 2021; Kano et al., 2009; Navarrete & Araque, 2010), a proportion of CB1 receptors is expressed in postsynaptic somatodendritic compartments in different brain areas including somatic sensory cortex (Bacci et al., 2004; Marinelli et al., 2008; Maroso et al., 2016). We showed that selective inhibition of neurosteroid synthesis in the recorded neuron blunted tonic GABA potentiation, suggesting that eCBs may act in an autocrine manner on somatodendritic CB1 receptors to transiently increase GABA tone via postsynaptic neurosteroid production (Fig. 11). This would explain the rapid onset and termination of the extrasynaptic GABA augmentation with respect to the classical retrograde synaptic GABA inhibition following depolarisation performed by eCB signalling, which occurred for tens of seconds with the maximum suppression occurring between 5 and 15 s from the end of the depolarisation step. In this scenario, the fast onset may reflect autocrine eCB action, whereas the rapid recovery may arise from neurosteroid negative‑feedback mechanisms (Midzak et al., 2011). However, most of the recorded cells displayed, with different magnitude, depolarisation‐induced tonic GABA augmentation and this is in contrast to the observation that only a minority of CB1 receptors are expressed postsynaptically.

It is also possible to hypothesise that either presynaptic or astrocytic CB1 receptors may trigger neurosteroid synthesis to induce extrasynaptic GABA_A_ potentiation (Fig. 11). Indeed, bath application of the neurosteroidogenesis inhibitor reduced the tonic current potentiation more effectively than the selective neurosteroidogenesis inhibition of postsynaptic neurons. This may suggest that retrograde eCB signalling may recruit neurosteroid synthesis in presynaptic terminals or astrocytes. Once synthesised, neurosteroids are probably released in a paracrine fashion to act on postsynaptic GABA_A_ receptor targets.

However, we cannot rule out the possibility that eCB signalling and neurosteroid actions on tonic GABA potentiation may occur without a causal relationship. Indeed, increased intracellular Ca^2+^, which occurs during sustained depolarisation, is a powerful stimulus for neurosteroid production (Guarneri et al., 1998; Kimoto et al., 2001). This would lead to depolarisation‐induced, CB1‐independent, extrasynaptic GABA_A_ receptor potentiation by autocrine neurosteroid action. Conversely, a neurosteroid‑independent, CB1‑mediated mechanism is difficult to reconcile with current evidence: CB1 receptors do not directly modulate extrasynaptic GABA_A_ receptors and presynaptic CB1 activation by reducing synaptic GABA release would simultaneously limit, rather than enhance, GABA spillover capable of engaging extrasynaptic receptors. Few studies have reported a functional interaction between GAT‐1 and CB1 receptor (Maneuf et al., 1996; Venderova et al., 2005), and in the neocortex both GAT‐1 and CB1 receptors are expressed perisynaptically in GABAergic terminals (Bodor et al., 2005; Conti et al., 2004). For these reasons it is possible that CB1 receptors, once activated, might be able to inhibit GAT‐1 activity, thus enhancing GABA spillover out of the synapse. However, GAT‑1 blockade did not occlude depolarisation‑induced tonic potentiation, arguing against a CB1–GAT‑1 interaction. Another mechanism by which eCB signalling might potentiate GABA tone independently from neurosteroid action is by increasing ambient GABA concentration through astrocytes. Indeed, several studies have reported that both astrocytes and microglial cells may synthesise and release GABA, contributing to extrasynaptic GABA tone (Kwak et al., 2020; Lee et al., 2010), and CB1 receptors are expressed in astrocytes, where they stimulate the release of gliotransmitters (Navarrete & Araque, 2008, 2010). However, experimentally elevating extracellular GABA did not occlude tonic potentiation, ruling out an astrocytic GABA source.

Among eCBs, 2‑AG is generally considered the principal activity‑dependent messenger, whereas AEA exerts a more tonic control over synaptic transmission. For this reason, our pharmacological manipulations focused primarily on the role of 2‑AG. The observation that DO34 strongly blunted, but did not abolish, *I*_GABA_ PD raises the possibility that AEA may contribute to this form of plasticity. Indeed, endogenous AEA tone is known to gate inhibitory plasticity across several brain regions, including the amygdala (Azad et al., 2004; Colangeli et al., 2020), striatum (Ade & Lovinger, 2007; Robbe et al., 2002), hippocampus (Colangeli et al., 2017; Kim & Alger, 2010) and neocortex (De‑May & Ali, 2013).

It is worth noting that 2‐AG has been postulated to directly bind GABA receptors (Baur et al., 2013; Sigel et al., 2011), and to synergise with neurosteroids to activate extrasynaptic GABA_A_ (Sigel et al., 2011). Thus, 2‑AG may also potentiate tonic inhibition through a CB1‑independent mechanism, potentially explaining why neither CB1 antagonism nor neurosteroidogenesis inhibition fully prevented tonic potentiation, since a residual tonic current might be provoked by the direct action of 2‐AG on extrasynaptic GABA_A_ receptors.

Taken together, these data provide evidence of a mechanistic link between endocannabinoid signalling, CB1 receptor activation and neurosteroid signalling in a transient form of tonic GABAergic plasticity. The combination of a fast retrograde eCB signal with the spatial and temporal precision of intracellular neurosteroid action provides a rapid, reversible tuning of pyramidal cell inhibitory tone. It has been reported that even small changes in tonic GABA conductance can propagate to large‑scale circuits, shifting anxiety‑related behaviours, altering sensory discrimination or driving pathological hypersynchrony (Botta et al., 2015; Cope et al., 2009; Kwak et al., 2020). Thus, the transient plasticity of tonic GABAergic inhibition described here may provide a mechanism capable of influencing excitability on behaviourally relevant timescales. As an activity‑dependent gain‑control mechanism, it can transiently suppress excessive firing and pathological hypersynchrony. Moreover, rapidly restoring a permissive excitability window may support stimulus‑dependent forms of plasticity that require precise temporal control of neuronal gain. In addition, by modulating the inhibitory tone of recently active pyramidal neurons, *I*_GABA_ PD can set the threshold for ensemble recruitment during cortical oscillation, thereby shaping the composition of cortical assemblies.

## Additional information

## Competing interests

The authors have no competing interests.

## Author contributions

R.C. and F.C. conceived the study. R.C. designed experiments, acquired data and wrote the manuscript. F.C. acquired funding, discussed the results and wrote the manuscript. Both authors have approved the final version of the manuscript and agree to be accountable for all aspects of the work in ensuring that questions related to the accuracy or integrity of any part of the work are appropriately investigated and resolved. All persons designated as authors qualify for authorship, and all those who qualify for authorship are listed. The experiments for this study were performed at the Department of Experimental and Clinical Medicine at the Università Politecnica delle Marche.

## Funding

This work was supported by funds granted by PRIN 2022BZWEKA to F.C.

## Author's present address

R. Colangeli: Departmental Faculty of Medicine and Surgery, UniCamillus‐Saint Camillus International University of Health Sciences, 00131 Rome, Italy.

F. Conti: European Brain Research Institute (EBRI) Rita Levi‐Montalcini, Viale Regina Elena 295, 00161 Rome, Italy.

## Supporting information


Peer Review History

## Data Availability

All data supporting the results presented in the paper are included in the figures.

## References

[tjp70624-bib-0001] Ade, K. K. , & Lovinger, D. M. (2007). Anandamide regulates postnatal development of long‐term synaptic plasticity in the rat dorsolateral striatum. The Journal of Neuroscience, 27(9), 2403–2409. 17329438 10.1523/JNEUROSCI.2916-06.2007 PMC6673491

[tjp70624-bib-0002] Anstee, Q. M. , Knapp, S. , Maguire, E. P. , Hosie, A. M. , Thomas, P. , Mortensen, M. , Bhome, R. , Martinez, A. , Walker, S. E. , Dixon, C. I. , Ruparelia, K. , Montagnese, S. , Kuo, Y. T. , Herlihy, A. , Bell, J. D. , Robinson, I. , Guerrini, I. , McQuillin, A. , Fisher, E. M. , … Thomas, H. C. (2013). Mutations in the Gabrb1 gene promote alcohol consumption through increased tonic inhibition. Nature Communications, 4, 2816. 10.1038/ncomms3816 PMC3843143 24281383

[tjp70624-bib-0003] Azad, S. C. , Monory, K. , Marsicano, G. , Cravatt, B. F. , Lutz, B. , Zieglgänsberger, W. , & Rammes, G. (2004). Circuitry for associative plasticity in the amygdala involves endocannabinoid signaling. The Journal of Neuroscience, 24(44), 9953–9961. 15525780 10.1523/JNEUROSCI.2134-04.2004 PMC6730232

[tjp70624-bib-0004] Bacci, A. , Huguenard, J. R. , & Prince, D. A. (2004). Long‐lasting self‐inhibition of neocortical interneurons mediated by endocannabinoids. Nature, 431(7006), 312–316. 15372034 10.1038/nature02913

[tjp70624-bib-0005] Bai, D. , Zhu, G. , Pennefather, P. , Jackson, M. F. , MacDonald, J. F. , & Orser, B. A. (2001). Distinct functional and pharmacological properties of tonic and quantal inhibitory postsynaptic currents mediated by γ‑aminobutyric acid receptors in hippocampal neurons. Molecular Pharmacology, 59(4), 814–824. 11259626 10.1124/mol.59.4.814

[tjp70624-bib-0006] Bakas, T. , van Nieuwenhuijzen, P. S. , Devenish, S. O. , McGregor, I. S. , Arnold, J. C. , & Chebib, M. (2017). The direct actions of cannabidiol and 2‐arachidonoyl glycerol at GABA(A) receptors. Pharmacological Research, 119, 358–370. 28249817 10.1016/j.phrs.2017.02.022

[tjp70624-bib-0007] Baur, R. , Kielar, M. , Richter, L. , Ernst, M. , Ecker, G. F. , & Sigel, E. (2013). Molecular analysis of the site for 2‐arachidonylglycerol (2‐AG) on the β_2_ subunit of GABA(A) receptors. Journal of Neurochemistry, 126(1), 29–36. 23600744 10.1111/jnc.12270

[tjp70624-bib-0008] Bodor, A. L. , Katona, I. , Nyíri, G. , Mackie, K. , Ledent, C. , Hájos, N. , & Freund, T. F. (2005). Endocannabinoid signaling in rat somatosensory cortex: Laminar differences and involvement of specific interneuron types. The Journal of Neuroscience, 25(29), 6845–6856. 16033894 10.1523/JNEUROSCI.0442-05.2005 PMC6725346

[tjp70624-bib-0009] Belelli, D. , & Lambert, J. J. (2005). Neurosteroids: Endogenous regulators of the GABA_A_ receptor. Nature Reviews Neuroscience, 6(7), 565–575. 15959466 10.1038/nrn1703

[tjp70624-bib-0010] Belelli, D. , Harrison, N. L. , Maguire, J. , Macdonald, R. L. , Walker, M. C. , & Cope, D. W. (2009). Extrasynaptic GABAA receptors: Form, pharmacology, and function. The Journal of Neuroscience, 29(41), 12757–12763. 19828786 10.1523/JNEUROSCI.3340-09.2009 PMC2784229

[tjp70624-bib-0011] Botta, P. , Demmou, L. , Kasugai, Y. , Markovic, M. , Xu, C. , Fadok, J. P. , Lu, T. , Poe, M. M. , Xu, L. , Cook, J. M. , Rudolph, U. , Sah, P. , Ferraguti, F. , & Lüthi, A. (2015). Regulating anxiety with extrasynaptic inhibition. Nature Neuroscience, 18(10), 1493–1500. 26322928 10.1038/nn.4102 PMC4607767

[tjp70624-bib-0012] Bragina, L. , Marchionni, I. , Omrani, A. , Cozzi, A. , Pellegrini‐Giampietro, D. E. , Cherubini, E. , & Conti, F. (2008). GAT‐1 regulates both tonic and phasic GABA(A) receptor‐mediated inhibition in the cerebral cortex. Journal of Neurochemistry, 105(5), 1781–1793. 18248614 10.1111/j.1471-4159.2008.05273.x

[tjp70624-bib-0013] Carver, C. M. , & Reddy, D. S. (2013). Neurosteroid interactions with synaptic and extrasynaptic GABA(A) receptors: Regulation of subunit plasticity, phasic and tonic inhibition, and neuronal network excitability. Psychopharmacology, 230(2), 151–188. 24071826 10.1007/s00213-013-3276-5 PMC3832254

[tjp70624-bib-0014] Castillo, P. E. , Chiu, C. Q. , & Carroll, R. C. (2011). Long‐term plasticity at inhibitory synapses. Current Opinion in Neurobiology, 21(2), 328–338. 21334194 10.1016/j.conb.2011.01.006 PMC3092861

[tjp70624-bib-0015] Cherubini, E. (2001). Generating diversity at GABAergic synapses. Trends in Neurosciences, 24(3), 155–162. 11182455 10.1016/s0166-2236(00)01724-0

[tjp70624-bib-0016] Chevaleyre, V. , & Castillo, P. E. (2003). Heterosynaptic LTD of hippocampal GABAergic synapses: A novel role of endocannabinoids in regulating excitability. Neuron, 38(3), 461–472. 12741992 10.1016/s0896-6273(03)00235-6

[tjp70624-bib-0017] Chevaleyre, V. , Takahashi, K. A. , & Castillo, P. E. (2006). Endocannabinoid‐mediated synaptic plasticity in the CNS. Annual Review of Neuroscience, 29, 37–76. 10.1146/annurev.neuro.29.051605.112834 16776579

[tjp70624-bib-0018] Colangeli, R. , Pierucci, M. , Benigno, A. , Campiani, G. , Butini, S. , & Di Giovanni, G. (2017). The FAAH inhibitor URB597 suppresses hippocampal maximal dentate afterdischarges and restores seizure‐induced impairment of short‐ and long‐term synaptic plasticity. Scientific Reports, 7(1), 11152. 28894217 10.1038/s41598-017-11606-1 PMC5593993

[tjp70624-bib-0019] Colangeli, R. , Morena, M. , Pittman, Q. J. , Hill, M. N. , & Teskey, G. C. (2020). Anandamide signaling augmentation rescues amygdala synaptic function and comorbid emotional alterations in a model of epilepsy. The Journal of Neuroscience, 40(31), 6068–6081. 32601243 10.1523/JNEUROSCI.0068-20.2020 PMC7392500

[tjp70624-bib-0020] Colangeli, R. , Morena, M. , Werner, A. , Thompson, R. J. , van der Stelt, M. , Pittman, Q. J. , Hill, M. N. , & Teskey, G. C. (2023). 2‐AG‐mediated control of GABAergic signaling is impaired in a model of epilepsy. The Journal of Neuroscience, 43(4), 571–583. 36460464 10.1523/JNEUROSCI.0541-22.2022 PMC9888507

[tjp70624-bib-0021] Colangeli, R. , Teskey, G. C. , & Di, G. G. (2021). Endocannabinoid‐serotonin systems interaction in health and disease. Progress in Brain Research, 259, 83–134. 33541682 10.1016/bs.pbr.2021.01.003

[tjp70624-bib-0022] Conti, F. , Minelli, A. , & Melone, M. (2004). GABA transporters in the mammalian cerebral cortex: Localization, development and pathological implications. Brain Research Reviews, 45(3), 196–212. 15210304 10.1016/j.brainresrev.2004.03.003

[tjp70624-bib-0023] Cope, D. W. , Hughes, S. W. , & Crunelli, V. (2005). GABAA receptor‐mediated tonic inhibition in thalamic neurons. The Journal of Neuroscience, 25(50), 11553–11563. 16354913 10.1523/JNEUROSCI.3362-05.2005 PMC6726040

[tjp70624-bib-0024] Cope, D. W. , Di Giovanni, G. , Fyson, S. J. , Orbán, G. , Errington, A. C. , Lőrincz, M. L. , Gould, T. M. , Carter, D. A. , & Crunelli, V. (2009). Enhanced tonic GABAA inhibition in typical absence epilepsy. Nature Medicine, 15(12), 1392–1398. 10.1038/nm.2058 PMC2824149 19966779

[tjp70624-bib-0025] De‐May, H. S. , & Ali, A. B. (2013). Perisomatic targeting interneurons show cell‐type specific DSI in neocortex. Journal of Neurophysiology, 109(Pt 1), 1219–1228.

[tjp70624-bib-0026] Farrant, M. , & Nusser, Z. (2005). Variations on an inhibitory theme: Phasic and tonic activation of GABA(A) receptors. Nature Reviews Neuroscience, 6(3), 215–229. 15738957 10.1038/nrn1625

[tjp70624-bib-0027] Fattorini, G. , Melone, M. , & Conti, F. (2020). A reappraisal of GAT‐1 localization in neocortex. Frontiers in Cellular Neuroscience, 14, 9. 32116556 10.3389/fncel.2020.00009 PMC7031676

[tjp70624-bib-0028] Földy, C. , Neu, A. , Jones, M. V. , & Soltesz, I. (2006). Presynaptic, activity‐dependent modulation of cannabinoid type 1 receptor‐mediated inhibition of GABA release. Journal of Neuroscience, 26(5), 1465–1469. 16452670 10.1523/JNEUROSCI.4587-05.2006 PMC6675496

[tjp70624-bib-0029] Gom, R. C. , George, A. G. , Harris, S. A. , Wickramarachchi, P. , Bhatt, D. , Acharjee, S. , Pittman, Q. J. , Hill, M. N. , Colangeli, R. , & Teskey, G. C. (2024). Emotional comorbidities in epilepsy result from seizure‐induced corticosterone activity. Neurobiology of Stress, 33, 100678. 39497812 10.1016/j.ynstr.2024.100678 PMC11533717

[tjp70624-bib-0030] Guarneri, P. , Russo, D. , Cascio, C. , De Leo, G. , Piccoli, F. , & Guarneri, R. (1998). Induction of neurosteroid synthesis by NMDA receptors in isolated rat retina: A potential early event in excitotoxicity. European Journal of Neuroscience, 10(5), 1752–1763. 9751147 10.1046/j.1460-9568.1998.00191.x

[tjp70624-bib-0031] Hebert‐Chatelain, E. , Desprez, T. , Serrat, R.á. , Bellocchio, L. , Soria‐Gomez, E. , Busquets‐Garcia, A. , Pagano Zottola, A. C. , Delamarre, A. , Cannich, A. , Vincent, P. , Varilh, M. , Robin, L. M. , Terral, G. , García‐Fernández, M. D. , Colavita, M. , Mazier, W. , Drago, F. , Puente, N. , Reguero, L. , … Marsicano, G. (2016). A cannabinoid link between mitochondria and memory. Nature, 539(7630), 555–559. 27828947 10.1038/nature20127

[tjp70624-bib-0032] Hoerbelt, P. , Lindsley, T. A. , & Fleck, M. W. (2015). Dopamine directly modulates GABAA receptors. The Journal of Neuroscience, 35(8), 3525–3536. 25716851 10.1523/JNEUROSCI.4390-14.2015 PMC6605556

[tjp70624-bib-0033] Kano, M. , Ohno‐Shosaku, T. , Hashimotodani, Y. , Uchigashima, M. , & Watanabe, M. (2009). Endocannabinoid‐mediated control of synaptic transmission. Physiological Reviews, 89(1), 309–380. 19126760 10.1152/physrev.00019.2008

[tjp70624-bib-0034] Katona, I. , & Freund, T. F. (2012). Multiple functions of endocannabinoid signaling in the brain. Annual Review of Neuroscience, 35, 529–558. 10.1146/annurev-neuro-062111-150420 PMC4273654 22524785

[tjp70624-bib-0035] Kim, J. , & Alger, B. E. (2010). Reduction in endocannabinoid tone is a homeostatic mechanism for specific inhibitory synapses. Nature Neuroscience, 13(5), 592–600. 20348918 10.1038/nn.2517 PMC2860695

[tjp70624-bib-0036] Kimoto, T. , Tsurugizawa, T. , Ohta, Y. , Makino, J. , Tamura, H. , Hojo, Y. , Takata, N. , & Kawato, S. (2001). Neurosteroid synthesis by cytochrome p450‐containing systems localized in the rat brain hippocampal neurons: N‐methyl‐D‐aspartate and calcium‐dependent synthesis. Endocrinology, 142(8), 3578–3589. 11459806 10.1210/endo.142.8.8327

[tjp70624-bib-0037] Khawaled, R. , Bruening‐Wright, A. , Adelman, J. P. , & Maylie, J. (1999). Bicuculline block of small‐conductance calcium‐activated potassium channels. Journal De Physiologie, 518(3), 415–424. 10.1007/s004240050915 10398861

[tjp70624-bib-0038] Kullmann, D. M. , Moreau, A. W. , Bakiri, Y. , & Nicholson, E. (2012). Plasticity of inhibition. Neuron, 75(6), 951–962. 22998865 10.1016/j.neuron.2012.07.030

[tjp70624-bib-0039] Kwak, H. , Koh, W. , Kim, S. , Song, K. , Shin, J. I. , Lee, J. M. , Lee, E. H. , Bae, J. Y. , Ha, G. E. , Oh, J. E. , Park, Y. M. , Kim, S. , Feng, J. , Lee, S. E. , Choi, J. W. , Kim, K. H. , Kim, Y. S. , Woo, J. , Lee, D. , … Cheong, E. (2020). Astrocytes control sensory acuity via tonic inhibition in the thalamus. Neuron, 108(4), 691–706.e10. 32905785 10.1016/j.neuron.2020.08.013

[tjp70624-bib-0040] Lee, S. , Yoon, B. E. , Berglund, K. , Oh, S. J. , Park, H. , Shin, H. S. , Augustine, G. J. , & Lee, C. J. (2010). Channel‐mediated tonic GABA release from glia. Science, 330(6005), 790–796. 20929730 10.1126/science.1184334

[tjp70624-bib-0041] Lee, V. , MacKenzie, G. , Hooper, A. , & Maguire, J. (2016). Reduced tonic inhibition in the dentate gyrus contributes to chronic stress‐induced impairments in learning and memory. Hippocampus, 26(10), 1276–1290. 27163381 10.1002/hipo.22604 PMC5030140

[tjp70624-bib-0042] Liu, Z. P. , Song, C. , Wang, M. , He, Y. , Xu, X. B. , Pan, H. Q. , Chen, W. B. , Peng, W. J. , & Pan, B. X. (2014). Chronic stress impairs GABAergic control of amygdala through suppressing the tonic GABAA receptor currents. Molecular Brain, 7(10), 32. 24758222 10.1186/1756-6606-7-32 PMC4012764

[tjp70624-bib-0043] Maguire, J. , & Mody, I. (2007). Neurosteroid synthesis‐mediated regulation of GABA(A) receptors: Relevance to the ovarian cycle and stress. The Journal of Neuroscience, 27(9), 2155–2162. 17329412 10.1523/JNEUROSCI.4945-06.2007 PMC6673487

[tjp70624-bib-0044] Maguire, J. , & Mody, I. (2008). GABA(A)R plasticity during pregnancy: Relevance to postpartum depression. Neuron, 59(2), 207–213. 18667149 10.1016/j.neuron.2008.06.019 PMC2875248

[tjp70624-bib-0045] Maguire, J. L. , Stell, B. M. , Rafizadeh, M. , & Mody, I. (2005). Ovarian cycle‐linked changes in GABA(A) receptors mediating tonic inhibition alter seizure susceptibility and anxiety. Nature Neuroscience, 8(6), 797–804. 15895085 10.1038/nn1469

[tjp70624-bib-0046] Makara, J. K. , Katona, I. , Nyíri, G. , Németh, B. , Ledent, C. , Watanabe, M. , de Vente, J. , Freund, T. F. , & Hájos, N. (2007). Involvement of nitric oxide in depolarization‐induced suppression of inhibition in hippocampal pyramidal cells during activation of cholinergic receptors. The Journal of Neuroscience, 27(38), 10211–10222. 17881527 10.1523/JNEUROSCI.2104-07.2007 PMC6672656

[tjp70624-bib-0047] Maneuf, Y. P. , Nash, J. E. , Crossman, A. R. , & Brotchie, J. M. (1996). Activation of the cannabinoid receptor by Δ9‐tetrahydrocannabinol reduces γ‐aminobutyric acid uptake in the globus pallidus. European Journal of Pharmacology, 308(2), 161–164. 8840127 10.1016/0014-2999(96)00326-3

[tjp70624-bib-0048] Marchionni, I. , Omrani, A. , & Cherubini, E. (2007). In the developing rat hippocampus a tonic GABA_A_‐mediated conductance selectively enhances the glutamatergic drive of principal cells. The Journal of Physiology, 581(Pt 2), 515–528. 17317750 10.1113/jphysiol.2006.125609 PMC2075167

[tjp70624-bib-0049] Marinelli, S. , Pacioni, S. , Bisogno, T. , Di Marzo, V. , Prince, D. A. , Huguenard, J. R. , & Bacci, A. (2008). The endocannabinoid 2‐arachidonoylglycerol is responsible for the slow self‐inhibition in neocortical interneurons. The Journal of Neuroscience, 28(50), 13532–13541. 19074027 10.1523/JNEUROSCI.0847-08.2008 PMC2615383

[tjp70624-bib-0050] Maroso, M. , Szabo, G. G. , Kim, H. K. , Alexander, A. , Bui, A. D. , Lee, S. H. , Lutz, B. , & Soltesz, I. (2016). Cannabinoid control of learning and memory through HCN channels. Neuron, 89(5), 1059–1073. 26898775 10.1016/j.neuron.2016.01.023 PMC4777634

[tjp70624-bib-0051] Melón, L. C. , Nolan, Z. T. , Colar, D. , Moore, E. M. , & Boehm, S. L., 2nd (2017). Activation of extrasynaptic δ‐GABA(A) receptors globally or within the posterior‐VTA has estrous‐dependent effects on consumption of alcohol and estrous‐independent effects on locomotion. Hormones and Behavior, 95, 65–75. 28765080 10.1016/j.yhbeh.2017.07.015 PMC5623082

[tjp70624-bib-0052] Melone, M. , Ciappelloni, S. , & Conti, F. (2015). A quantitative analysis of cellular and synaptic localization of GAT‐1 and GAT‐3 in rat neocortex. Brain Structure and Function, 220(2), 885–897. 24368619 10.1007/s00429-013-0690-8

[tjp70624-bib-0053] Midzak, A. , Akula, N. , Lecanu, L. , & Papadopoulos, V. (2011). Novel androstenetriol interacts with the mitochondrial translocator protein and controls steroidogenesis. Journal of Biological Chemistry, 286(11), 9875–9887. 21209087 10.1074/jbc.M110.203216 PMC3058962

[tjp70624-bib-0054] Minelli, A. , Brecha, N. C. , Karschin, C. , DeBiasi, S. , & Conti, F. (1995). GAT‐1, a high‐affinity GABA plasma membrane transporter, is localized to neurons and astroglia in the cerebral cortex. The Journal of Neuroscience, 15(11), 7734–7746. 7472524 10.1523/JNEUROSCI.15-11-07734.1995 PMC6578038

[tjp70624-bib-0055] Minelli, A. , DeBiasi, S. , Brecha, N. C. , Vitellaro Zuccarello, L. , & Conti, F. (1996). GAT‐3, a high‐affinity GABA plasma membrane transporter, is localized to astrocytic processes, and it is not confined to the vicinity of GABAergic synapses in the cerebral cortex. The Journal of Neuroscience, 16(19), 6255–6264. 8815906 10.1523/JNEUROSCI.16-19-06255.1996 PMC6579190

[tjp70624-bib-0056] Mody, I. , & Pearce, R. A. (2004). Diversity of inhibitory neurotransmission through GABA(A) receptors. Trends in Neurosciences, 27(9), 569–575. 15331240 10.1016/j.tins.2004.07.002

[tjp70624-bib-0057] Navarrete, M. , & Araque, A. (2008). Endocannabinoids mediate neuron‐astrocyte communication. Neuron, 57(6), 883–893. 18367089 10.1016/j.neuron.2008.01.029

[tjp70624-bib-0058] Navarrete, M. , & Araque, A. (2010). Endocannabinoids potentiate synaptic transmission through stimulation of astrocytes. Neuron, 68(1), 113–126. 20920795 10.1016/j.neuron.2010.08.043

[tjp70624-bib-0059] Raux, P. L. , & Vallée, M. (2023). Cross‐talk between neurosteroid and endocannabinoid systems in cannabis addiction. Journal of Neuroendocrinology, 35(2), e13191. 36043319 10.1111/jne.13191

[tjp70624-bib-0060] Robbe, D. , Kopf, M. , Remaury, A. , Bockaert, J. , & Manzoni, O. J. (2002). Endogenous cannabinoids mediate long‐term synaptic depression in the nucleus accumbens. Proceedings of the National Academy of Sciences, 99(12), 8384–8388. 10.1073/pnas.122149199 PMC123076 12060781

[tjp70624-bib-0061] Semyanov, A. , Walker, M. C. , & Kullmann, D. M. (2003). GABA uptake regulates cortical excitability via cell type‐specific tonic inhibition. Nature Neuroscience, 6(5), 484–490. 12679782 10.1038/nn1043

[tjp70624-bib-0062] Semyanov, A. , Walker, M. C. , Kullmann, D. M. , & Silver, R. A. (2004). Tonically active GABA A receptors: Modulating gain and maintaining the tone. Trends in Neurosciences, 27(5), 262–269. 15111008 10.1016/j.tins.2004.03.005

[tjp70624-bib-0063] Sigel, E. , Baur, R. , Rácz, I. , Marazzi, J. , Smart, T. G. , Zimmer, A. , & Gertsch, J.ü (2011). The major central endocannabinoid directly acts at GABA(A) receptors. Proceedings of the National Academy of Sciences, 108(44), 18150–18155. 10.1073/pnas.1113444108 PMC3207709 22025726

[tjp70624-bib-0064] Soltesz, I. , Alger, B. E. , Kano, M. , Lee, S. H. , Lovinger, D. M. , Ohno‐Shosaku, T. , & Watanabe, M. (2015). Weeding out bad waves: Towards selective cannabinoid circuit control in epilepsy. Nature Reviews Neuroscience, 16(5), 264–277. 25891509 10.1038/nrn3937 PMC10631555

[tjp70624-bib-0065] Stell, B. M. , & Mody, I. (2002). Receptors with different affinities mediate phasic and tonic GABA currents in hippocampal neurons. The Journal of Neuroscience, 22(10), RC223–RC223. 12006605 10.1523/JNEUROSCI.22-10-j0003.2002 PMC6757628

[tjp70624-bib-0066] Stell, B. M. , Brickley, S. G. , Tang, C. Y. , Farrant, M. , & Mody, I. (2003). Neuroactive steroids reduce neuronal excitability by selectively enhancing tonic inhibition mediated by delta subunit‐containing GABAA receptors. Proceedings of the National Academy of Sciences, 100(24), 14439–14444. 10.1073/pnas.2435457100 PMC283610 14623958

[tjp70624-bib-0067] Tanaka, M. , & Sokabe, M. (2012). Continuous de novo synthesis of neurosteroids is required for normal synaptic transmission and plasticity in the dentate gyrus of the rat hippocampus. Neuropharmacology, 62(7), 2373–2387. 22365983 10.1016/j.neuropharm.2012.02.007

[tjp70624-bib-0068] Tanimura, A. , Yamazaki, M. , Hashimotodani, Y. , Uchigashima, M. , Kawata, S. , Abe, M. , Kita, Y. , Hashimoto, K. , Shimizu, T. , Watanabe, M. , Sakimura, K. , & Kano, M. (2010). The endocannabinoid 2‐arachidonoylglycerol produced by diacylglycerol lipase alpha mediates retrograde suppression of synaptic transmission. Neuron, 65(3), 320–327. 20159446 10.1016/j.neuron.2010.01.021

[tjp70624-bib-0069] Ueno, S. , Bracamontes, J. , Zorumski, C. , Weiss, D. S. , & Steinbach, J. H. (1997). Bicuculline and gabazine are allosteric inhibitors of channel opening of the GABA receptor. The Journal of Neuroscience, 17(2), 625–634. 8987785 10.1523/JNEUROSCI.17-02-00625.1997 PMC6573228

[tjp70624-bib-0070] Urban‐Ciecko, J. , & Mozrzymas, J. W. (2011). Sex‐specificity of associative learning‐induced changes in GABAergic tonic inhibition in layer 4 neurons of mouse barrel cortex. Behavioural Brain Research, 219(2), 373–377. 21238501 10.1016/j.bbr.2011.01.013

[tjp70624-bib-0071] Vallée, M. , Vitiello, S. , Bellocchio, L. , Hébert‐Chatelain, E. , Monlezun, S. , Martin‐Garcia, E. , Kasanetz, F. , Baillie, G. L. , Panin, F. , Cathala, A. , Roullot‐Lacarrière, V. , Fabre, S. , Hurst, D. P. , Lynch, D. L. , Shore, D. M. , Deroche‐Gamonet, V. , Spampinato, U. , Revest, J. M. , Maldonado, R. , … Piazza, P. V. (2014). Pregnenolone can protect the brain from cannabis intoxication. Science, 343(6166), 94–98. 24385629 10.1126/science.1243985 PMC4057431

[tjp70624-bib-0072] Venderova, K. , Brown, T. M. , & Brotchie, J. M. (2005). Differential effects of endocannabinoids on [(3)H]‐GABA uptake in the rat globus pallidus. Experimental Neurology, 194(1), 284–287. 15899265 10.1016/j.expneurol.2005.02.012

[tjp70624-bib-0073] Yamada, J. , Furukawa, T. , Ueno, S. , Yamamoto, S. , & Fukuda, A. (2007). Molecular basis for the GABAA receptor–Mediated tonic inhibition in rat somatosensory cortex. Cerebral Cortex, 17(8), 1782–1787. 16997904 10.1093/cercor/bhl087

[tjp70624-bib-0074] Yoshida, T. , Hashimoto, K. , Zimmer, A. , Maejima, T. , Araishi, K. , & Kano, M. (2002). The cannabinoid CB1 receptor mediates retrograde signals for depolarization‐induced suppression of inhibition in cerebellar Purkinje cells. The Journal of Neuroscience, 22(5), 1690–1697. 11880498 10.1523/JNEUROSCI.22-05-01690.2002 PMC6758890

[tjp70624-bib-0075] Zurek, A. A. , Yu, J. , Wang, D. S. , Haffey, S. C. , Bridgwater, E. M. , Penna, A. , Lecker, I. , Lei, G. , Chang, T. , Salter, E. W. , & Orser, B. A. (2014). Sustained increase in α5GABAA receptor function impairs memory after anesthesia. Journal of Clinical Investigation, 124(12), 5437–5441. 25365226 10.1172/JCI76669 PMC4348961

