# Supplementary material for: The endogenous cannabinoid system gates plasticity of tonic GABA inhibition
Source: J Physiol. 2026 May 27;604(12):4984–5010. doi: 10.1113/JP289183 (PMC13267673; doi:10.1113/JP289183)
Supplement: Supplementary file 1 — Peer Review History [file TJP-604-4984-s001.pdf]

The Journal of Physiology

The endogenous cannabinoid system gates plasticity of tonic GABA inhibition

Roberto Colangeli and Fiorenzo Conti
DOI: 10.1113/JP289183

Corresponding author(s): Fiorenzo Conti (fiorenzo.conti@staff.univpm.it)

The following individual(s) involved in review of this submission have agreed to reveal their identity: Eric S Levine (Referee #2)

Review Timeline:

Submission Date:	29-Apr-2025
Editorial Decision:	10-Jun-2025
Revision Received:	15-Apr-2026
Accepted:	30-Apr-2026

Senior Editor: Nathan Schoppa

Reviewing Editor: Nathan Schoppa

Transaction Report:

(Note: With the exception of the correction of typographical or spelling errors that could be a source of ambiguity, letters and reports are not edited. Depending on transfer agreements, referee reports obtained elsewhere may or may not be included in this compilation. Referee reports are anonymous unless the Referee chooses to sign their reports.)

Dear Dr Conti,

Re: JP-RP-2025-289183 "**The endogenous cannabinoid system gates plasticity of tonic GABA inhibition**" by Roberto Colangeli and Fiorenzo Conti

Thank you for submitting your manuscript to The Journal of Physiology. It has been assessed by a Reviewing Editor and by 2 expert referees and we are pleased to tell you that it is potentially acceptable for publication following satisfactory major revision.

Please advise your co-authors of this decision as soon as possible.

The referee reports are copied at the end of this email.

Please address all the points raised and incorporate all requested revisions or explain in your Response to Referees why a change has not been made. We hope you will find the comments helpful and that you will be able to return your revised manuscript within 9 months. If you require longer than this, please contact journal staff: jp@physoc.org. Please note that this letter does not constitute a guarantee for acceptance of your revised manuscript.

Your revised manuscript should be submitted online using the link in your Author Tasks: Link Not Available. This link is accessible via your account as Corresponding Author; it is not available to your co-authors. If this presents a problem, please contact journal staff (jp@physoc.org). Image files from the previous version are retained on the system. Please ensure you replace or remove any files that are being revised.

If you do not wish to submit a revised version of your manuscript, you must inform our journal staff (jp@physoc.org) or reply to this email to request withdrawal. Please note that a manuscript must be formally withdrawn from the peer review process at one journal before it may be submitted to another journal.

TRANSPARENT PEER REVIEW POLICY: To improve the transparency of its peer review process, The Journal of Physiology publishes online as supporting information the peer review history of all articles accepted for publication. Readers will have access to decision letters, including Editors' comments and referee reports, for each version of the manuscript, as well as any author responses to peer review comments. Referees can decide whether or not they wish to be named on the peer review history document.

ABSTRACT FIGURES: Authors are expected to use The Journal's premium BioRender account to create/redraw their Abstract Figures. Information on how to access this account is here: https://physoc.onlinelibrary.wiley.com/journal/14697793/biorender-access.

This will enable Authors to create and download high-resolution figures. If authors have used the free BioRender service, they can use the instructions provided in the link above to download a high-resolution version suitable for publication.

The link provided should only be used for the purposes of this submission. Authors will be charged for figures created on this account if they are not related to this manuscript submission.

LANGUAGE EDITING AND SUPPORT FOR PUBLICATION: If you would like help with English language editing, or other article preparation support, Wiley Editing Services offers expert help, including English Language Editing, as well as translation, manuscript formatting, and figure formatting at www.wileyauthors.com/eeo/preparation. You can also find resources for Preparing Your Article for general guidance about writing and preparing your manuscript at www.wileyauthors.com/eeo/prepresources.

RIGOUR AND REPRODUCIBILITY: We recommend authors consult The Journal's Rigour and Reproducibility requirements to ensure that their manuscript is suitable for publication: https://physoc.onlinelibrary.wiley.com/pb-assets/hub-assets/physoc/documents/TJP-Rigour-and-Reproducibility-Requirements.pdf

REVISION CHECKLIST:

Check that your Methods section conforms to journal policy: https://jp.msubmit.net/cgi-bin/main.plex?form_type=display_requirements#methods.

Check that data presented conforms to the statistics policy: https://jp.msubmit.net/cgi-bin/main.plex?form_type=display_requirements#statistics.

Upload a full Response to Referees file. To create your 'Response to Referees': copy all the reports, including any comments from the Senior and Reviewing Editors, into a Microsoft Word, or similar, file and respond to each point, using font or background colour to distinguish comments and responses and upload as the required file type.

Please upload two versions of your manuscript text: one with all relevant changes highlighted and one clean version with no

changes tracked. The manuscript file should include all tables and figure legends, but each figure/graph should be uploaded as separate, high-resolution files.

You may also upload:

- 'Potential Cover Art' for consideration as the issue's cover image
- Appropriate Supporting Information (Video, audio or data set: see https://jp.msubmit.net/cgi-bin/main.plex?form_type=display_requirements#supp).

We look forward to receiving your revised submission.

If you have any queries, please reply to this email and we will be pleased to advise.

Yours sincerely,

Nathan Schoppa
Senior Editor
The Journal of Physiology

REQUIRED ITEMS

- The Journal of Physiology funds authors of provisionally accepted papers to use the premium BioRender site to create high resolution schematic figures. Follow this link and enter your details and the manuscript number to create and download figures. Upload these as the figure files for your revised submission. If you choose not to take up this offer, we require figures to be of similar quality and resolution. If you are opting out of this service to authors, state this in the Comments section on the Detailed Information page of the submission form. The link provided should only be used for the purposes of this submission. Authors will be charged for figures created on this premium BioRender account if they are not related to this manuscript submission.

- Please upload separate high-quality figure files via the submission form.

- Please ensure that the Article File you upload is a Word file.

EDITOR COMMENTS

Senior Editor:

Comments to the Author:
Your manuscript has been reviewed by two expert referees, who both felt that your study is reporting novel results around tonic inhibition and would be of general interest. Both referees did however raise a number of concerns that will require additional analysis and a few additional experiments to strengthen conclusions, along with some other changes in the text. All concerns that were raised should be addressed. Amongst the most important included:

1. If it is possible, the authors should conduct a few experiments with the CB1 antagonist AM251 in which Itonic is measured before and after AM251 in the same cell. This might help reveal some effects of the drug that were missed in the comparisons of Itonic measured in different cell populations.

2. The authors should confirm a few of their most important results based on the effect of bicuculline with the more selective GABA(A) blocker gabazine.

3. As suggested in a few points made by referee 2, the authors should show more of their data traces. This will help in assessing potential eCB-mediated DSI and also for confirming the accuracy of measuring tonic versus phasic inhibition. I

also had a concern with the difficulty of measuring tonic inhibition given the high frequency of the sIPSCs.

4. The authors should strengthen their conclusion around the role of 2-AG with experiments that address the role of AEA or tone down language about involvement of 2-AG.

5. The authors should consider doing additional experiments with the more selective CB1 antagonist NESS-0327 or at least discuss the limitations of AM-251.

6. Both reviewers cited grammatical and language errors and typos that will need to be addressed.

7. I appreciate that the authors provide values for the number of cells "n" and also the number "N" that I believe reflects the number of mice. The authors should clarify in the methods what exactly "n" and "N" are.

REFEREE COMMENTS

Referee #1:

This manuscript from Colangeli & Conti suggests that transient depolarization of excitatory cells in the S1 cortex results in an increase in tonic GABA currents, and it assesses mechanisms that may underlie this phenomenon. Using patch-clamp electrophysiology and pharmacology, the authors explore the role of endocannabinoid (eCB) signaling and steroid synthesis. They conclude that the 'post-depolarization potentiation' of tonic GABA requires both eCB signaling (suggesting the process is 2-AG and CB1R dependent) and de novo synthesis of steroids (as suggested by inhibition of the P450scc enzyme using AMG), though the precise cellular mechanisms remain unclear.

The key message of the study about the depolarization-induced increase in tonic GABA is innovative and interesting. The manuscript incorporates a variety of techniques and interesting experiments which hold great merit. However, there are some concerns with the manuscript's findings and conclusions, as discussed below.

Major:

1. Aside from the bicuculline experiments, most were conducted by comparing different cells from different slices, each incubated in the corresponding drug treatment group. While this approach is understandable given the slow time course of both AM251 and DO34, it would strengthen the paper to include a few cells in which Itonic and IGABA PD are assessed before and after AM251 treatment.

2. Is this process only 2-AG dependent, or does blocking anandamide (AEA) synthesis or breakdown similarly affect the phenomenon? I suggest the authors explore the role of AEA in this process or tone down the language on the exclusive dependence of 2-AG.

3. The neurosteroid argument relies entirely on AMG, which blocks cytochrome P450scc. Ideally, testing a specific neuromodulatory candidate would significantly strengthen the claim. If the authors do not have candidate neuromodulators, a more detailed discussion of other potential effects of cytochrome P450 inhibition would be useful.

4. The authors bring up astrocytes and microglia as possible sources of GABA in the results section, but do not directly investigate a link. I suggest moving this to the discussion section since it is largely an untested hypothesis.

Minor:

1. Please refer to your holding current calculation as something other than Ih as for most people, the latter refers to the hyperpolarization-activated h-current.

2. There are several grammatical errors and typos to be corrected throughout the manuscript.

Referee #2:

This manuscript reports that tonic GABA inhibition in cortical neurons can be transiently increased by a mechanism that requires endocannabinoid signaling and neurosteroid synthesis. This modulation of tonic inhibition is not accounted for by increased extracellular GABA concentration. This is an interesting and original finding and potentially represents a worthwhile contribution to the existing literature. However, there are several methodological and substantive points raised below that should be addressed to support the validity of the conclusions.

1 - It is reported that recordings were performed on layer 2/3 principal neurons. Does that mean pyramidal neurons, and how were these neurons identified?

2 - What was the final concentration of DMSO when drugs were applied to the slices, and was a DMSO control performed?

3 - Bicuculline was used to block GABA-A receptors to quantify tonic inhibition, however bicuculline is also known to block Ca-activated potassium channels, which could confound those measurements. Gabazine (SR-95531) is a widely available selective antagonist at GABA-A receptors that avoids this problem. At least some of the foundational experiments in the manuscript should be repeated with GABAzine to rule out effects on Ca-activated potassium channels.

4 - AM251 is used to block CB1 receptors but also acts as a CB1 inverse agonist. Why did the authors use AM-251 instead of NESS-0327, which is a potent antagonist of CB1 that is more selective for the CB1 receptor over CB2 than AM-251, and it is a purely neutral antagonist that does not act as an inverse agonist. The potential confounds of inverse agonism should be addressed in the Discussion and complementary experiments with NESS-0327 should be considered.

5 - It is stated in the Methods that "Studies were designed to generate equal-sized groups", however there are several experiments with very unequal n's between groups, e.g. Figs. 2, 3, and 4. This makes it difficult to support some of the conclusions, and probably relates to why data are presented as mean+SD, instead of the more common mean+SEM.

6 - There is no positive control for the negative result reported for the GAT-1 inhibitor NO711. Also, could the lack of effect of GAT-1 inhibition represent a ceiling effect?

7 - The authors should report whether the depolarization protocol also resulted in eCB-mediated DSI. The example sweeps that are shown are too short in duration to observe this, and this is an important point that should be addressed.

8 - Example sweeps at expanded time scales are needed to evaluate the quality of the recordings and accuracy of quantification. It is difficult to see whether tonic GABA inhibition was stable during the baseline period, and how it was accurately measured given the high frequency of phasic events. In addition, is the capacitance artifact after depolarization blanked out in the example sweeps? Because the altered tonic current is so brief, it is important to show unedited example sweeps.

9 - The authors should expand their discussion of the potential functional relevance of the altered tonic inhibition, especially given the extremely brief duration of the effect.

10 - How was Cm calculated?

11 - The manuscript needs to be thoroughly reviewed for grammatical errors, language errors, and numerous typos.

END OF COMMENTS

ANSWERS TO EDITOR' AND REVIEWERS' COMMENTS

Senior Editor:

Comments to the Author:
Your manuscript has been reviewed by two expert referees, who both felt that your study is reporting novel results around tonic inhibition and would be of general interest. Both referees did however raise a number of concerns that will require additional analysis and a few additional experiments to strengthen conclusions, along with some other changes in the text.

We thank you for the opportunity to revise our manuscript, and we are grateful to you and the referees for the constructive and insightful evaluation. We are pleased that the referees recognised the novelty and broad interest of our findings on tonic GABAergic inhibition. As requested, we have carefully addressed all concerns raised and performed the additional analyses and experiments necessary to strengthen the mechanistic conclusions of the study. We believe that these revisions have substantially improved the clarity, rigor, and overall impact of the manuscript.

All concerns that were raised should be addressed. Amongst the most important included:
1. If it is possible, the authors should conduct a few experiments with the CB1 antagonist AM251 in which I_{tonic} is measured before and after AM251 in the same cell. This might help reveal some effects of the drug that were missed in the comparisons of I_{tonic} measured in different cell populations.

We fully agree that this point is particularly important. In response, we performed new within-cell recordings in which the depolarisation-evoked current (ΔI_{hold}) was measured before and after AM251 in the same neuron. These experiments confirmed that AM251 significantly attenuates ΔI_{GABA} PD, thereby strengthening the conclusion that CB1 receptor signalling contributes to the depolarization-induced potentiation of tonic inhibition. As detailed in our response to Reviewer 1, the experimental sequence employed (ACSF → AM251 → BIC + AM251) allowed a direct pre- versus post-AM251 comparison without requiring multiple bicuculline wash-ins and wash-outs. In our preparation bicuculline wash out was slow and, without a focal rapid perfusion system, the prolonged sequence of drug exchanges often compromised the stability of the whole-cell configuration. This approach is not feasible for basal I_{tonic}, whose quantification necessarily requires bicuculline wash-out. For this reason, we focused the paired protocol on the depolarisation-evoked component of tonic inhibition, which is the central phenomenon under investigation.

2. The authors should confirm a few of their most important results based on the effect of bicuculline with the more selective GABA(A) blocker gabazine.

We totally agree with the Editor that the point raised by Reviewer 2 regarding the use of gabazine is essential for strengthening the manuscript. As requested, we repeated the key experiments with gabazine, which confirmed the $GABA_A$ receptor-dependence of the depolarisation-evoked tonic current. Moreover, the gabazine-sensitive I_{GABA} PD was attenuated by DO34, thus validating that the post-depolarization potentiation is mediated by

$GABA_A$ receptors and requires 2-AG mobilization. Thanks to the reviewer's concern we were able to reveal that the shift produced by bicuculline on basal tonic current is probably not mediated by extrasynaptic $GABA_A$ receptor activation since gabazine failed to reveal a basal tonic current.

Importantly, the discrepancy between bicuculline and gabazine regarding the basal tonic current intersected with a complementary concern raised by Reviewer 1, who suggested testing a specific neuromodulatory candidate to strengthen the neurosteroid-based interpretation. By integrating these two reviewer comments, we performed an additional experiment using allopregnanolone (ALLO) and gabazine. Under ALLO, gabazine, which had previously been unable to reveal any basal tonic conductance, unmasked a clear tonic current. This result demonstrates that gabazine is sensitive to both I_{GABA} PD and neurosteroid-dependent modulation of extrasynaptic GABA receptors, thus strongly supporting the conclusion that depolarisation might recruit a neurosteroid-dependent pathway. We believe that the gabazine + ALLO experiment provided an important mechanistic contribution that further strengthened the overall conclusions of the study.

3. As suggested in a few points made by referee 2, the authors should show more of their data traces. This will help in assessing potential eCB-mediated DSI and also for confirming the accuracy of measuring tonic versus phasic inhibition. I also had a concern with the difficulty of measuring tonic inhibition given the high frequency of the sIPSCs.

We acknowledge the importance of this point. We have substantially revised the figures and analyses. As detailed in our point-by-point responses, all representative examples now include both the full recording sweeps and expanded-timescale segments, allowing the reader to clearly assess baseline stability, the presence of DSI, and the distinction between tonic and phasic inhibition.

To directly address the reviewer's question about DSI, we performed a quantitative analysis of synaptic inhibition following the depolarizing step. As shown in the revised Fig. 1, the depolarization protocol reliably induced a transient suppression of inhibitory synaptic transmission, confirming the presence of DSI under our experimental conditions.

We also addressed the concern regarding the difficulty of measuring tonic inhibition in the presence of high-frequency sIPSCs. In addition to providing example sweeps at expanded timescale, we re-analysed the entire dataset using a more rigorous and conservative procedure. Mean I_{hold} was extracted from 5-s epochs by averaging 10-ms segments sampled every 100 ms while excluding data points corresponding to IPSC decay. Clean baseline distributions were then quantified using Gaussian fits of the amplitude histograms obtained before and after antagonist application (Cope et al., 2009). All statistics are now updated in the manuscript. This refined analytical approach provides a more accurate and reliable estimate of tonic GABA currents and directly addresses the reviewer's concern about the impact of phasic events on tonic current quantification.

Together, we believe that the addition of full sweeps, expanded-timescale traces, explicit visualisation of capacitive transients, and a more stringent analytical pipeline, substantially improves the transparency and robustness of the data presentation and fully address the issues

raised by Reviewer 2 and highlighted by the Editor.

4. The authors should strengthen their conclusion around the role of 2-AG with experiments that address the role of AEA or tone down language about involvement of 2-AG.

We do agree that AEA could in principle contribute to depolarisation-induced modulation of tonic GABA currents. In the present study, our pharmacological manipulation selectively targeted 2-AG synthesis, since, in many brain regions, including several cortical structures, DSI is mediated predominantly, and in some cases exclusively, by 2-AG. Moreover, AEA synthesis involves multiple parallel biosynthetic routes. Among the currently available inhibitors of AEA production, LEI-404 is an emerging good candidate compound used to study AEA signalling. However, since this compound inhibits the NAPE-PLD enzyme it would also inhibit a variety of neuroactive N-acylethanolamines that share the same biosynthetic pathways as AEA. These considerations provided the rationale for initially focusing on 2-AG–related pathway. Nevertheless, we have toned down the language about the specific eCB involved in this form of plasticity, replaced 2-AG with eCBs both in in the text and Fig.11, and acknowledged that our results rely on the pharmacological tool used.

5. The authors should consider doing additional experiments with the more selective CB1 antagonist NESS-0327 or at least discuss the limitations of AM-251.

We performed additional experiments using the more selective and neutral CB1 antagonist NESS-0327. NESS-0327 significantly reduced the post-depolarization potentiation of tonic inhibition, fully replicating the effect originally observed with AM251 and confirming that the phenomenon reflects genuine CB1 receptor blockade.

As part of the statistical revision raised by Reviewer 2 in a separate point, we re-analysed some of our dataset using variance-appropriate tests. In this re-analysis, we found that AM251 in the between-cell comparison was no longer significant, which further underscored the need for more rigorous pharmacological validation. Importantly, both the new NESS-0327 experiments and a paired AM251 protocol performed in the revision independently confirmed that CB1 signalling is involved in the depolarization-evoked enhancement of tonic inhibition. Overall, these additions provide a stronger and more rigorous demonstration of CB1 receptor involvement.

6. Both reviewers cited grammatical and language errors and typos that will need to be addressed.
We have revised the manuscript to correct grammatical errors, typos, and stylistic inconsistencies, improving clarity and readability.

7. I appreciate that the authors provide values for the number of cells "n" and also the number "N" that I believe reflects the number of mice. The authors should clarify in the methods what exactly "n" and "N" are.
We thank the Editor for this clarification request. In the revised manuscript, we have now explicitly defined throughout the text that n refers to the number of recorded cells ($n_{(cell)}$) and

N refers to the number of animals ($N_{(mice)}$) from which those cells were obtained. We hope this addition improves clarity for the reader.

REFEREE COMMENTS

Referee #1:

This manuscript from Colangeli & Conti suggests that transient depolarization of excitatory cells in the S1 cortex results in an increase in tonic GABA currents, and it assesses mechanisms that may underlie this phenomenon. Using patch-clamp electrophysiology and pharmacology, the authors explore the role of endocannabinoid (eCB) signaling and steroid synthesis. They conclude that the 'post-depolarization potentiation' of tonic GABA requires both eCB signaling (suggesting the process is 2-AG and CB1R dependent) and de novo synthesis of steroids (as suggested by inhibition of the P450scc enzyme using AMG), though the precise cellular mechanisms remain unclear.

The key message of the study about the depolarization-induced increase in tonic GABA is innovative and interesting. The manuscript incorporates a variety of techniques and interesting experiments which hold great merit. However, there are some concerns with the manuscript's findings and conclusions, as discussed below.

We thank the Reviewer for the positive assessment, and we appreciate the recognition of the conceptual relevance of our findings. The concerns raised are important and have prompted us to revise the manuscript extensively, perform additional analyses, and carry out new experiments that have substantially strengthened the conclusions. Below, we address each point raised by the Reviewer in a point-by-point manner.

Major:

1. Aside from the bicuculline experiments, most were conducted by comparing different cells from different slices, each incubated in the corresponding drug treatment group. While this approach is understandable given the slow time course of both AM251 and DO34, it would strengthen the paper to include a few cells in which I_{tonic} and I_{GABA} PD are assessed before and after AM251 treatment.

We thank the Reviewer for this helpful suggestion. We agree that within-cell comparisons provide a powerful and sensitive approach to assess drug effects on tonic GABA currents. In response, we performed a paired experiment in which the depolarisation-evoked current (ΔI_{hold}) was measured in the same neuron before and after AM251 application and subsequently after BIC application. To compute the pre-AM251 and post-AM251 I_{GABA} PD values, both pre-AM251 and post AM251 ΔI_{hold} values were normalised to the same bicuculline-sensitive component. This experiment confirmed that AM251 significantly attenuates I_{GABA} PD, thereby strengthening the conclusion that CB1 receptor signalling contributes to the depolarisation-induced potentiation of tonic inhibition. Moreover, this

experimental design (ACSF → AM251 → BIC + AM251) allowed a direct pre- versus post-AM251 comparison without requiring multiple bicuculline wash-ins and wash-outs. In our experimental conditions, bicuculline wash-out was slow, and without rapid focal perfusion systems, which are not available in our setup, the prolonged sequence of drug exchanges often compromised the stability of the whole-cell configuration. Since the within cell approach used for I_{GABA} PD, is not feasible for basal I_{tonic}, whose quantification necessarily requires BIC washout, we decided to focus the within cell experiment on I_{GABA} PD, the central phenomenon under investigation. In addition, this within-cell strategy used for AM251 is consistent with the approach we had already adopted in the originally submitted version of the manuscript to assess the effect of exogenously applied GABA on I_{GABA} PD, and with the protocol used in the revised manuscript to evaluate the effect of allopregnanolone. We believe that, by avoiding repeated bicuculline applications, this design preserves recording stability while providing a robust and reliable paired comparison within the same neuron. We hope that this new within-cell evidence satisfactorily addresses the Reviewer's request.

2. Is this process only 2-AG dependent, or does blocking anandamide (AEA) synthesis or breakdown similarly affect the phenomenon? I suggest the authors explore the role of AEA in this process or tone down the language on the exclusive dependence of 2-AG.

We fully agree that both major endocannabinoids, 2-AG and anandamide (AEA), could in principle contribute to depolarisation-induced modulation of tonic GABA currents. In the present study, our pharmacological manipulations selectively targeted 2-AG synthesis because in many brain regions, including several cortical structures, DSI is mediated predominantly, and in some cases exclusively, by 2-AG. Moreover, AEA synthesis involves multiple parallel biosynthetic routes that provide compensatory mechanisms when attempts are made to inhibit AEA production. We are aware that a compound that reduces AEA does exist, the NAPE-PLD inhibitor LEI-401; however, this compound also affects a variety of N-acylethanolamines that share the same biosynthetic pathways as AEA (Mock, *et al. Nat Chem Biol;* 2020). These considerations provided the rationale for initially focusing on the 2-AG–related pathways.

That said, we do not rule out the possibility that AEA may also contribute to the depolarisation-induced enhancement of tonic inhibition. In response to the Reviewer's suggestion, we have revised the text to avoid implying exclusive dependence on 2-AG and to clarify that our conclusions reflect the specific pharmacological tools employed. We now explicitly state that the potential involvement of AEA remains an open and interesting question for future work.

We hope that these clarifications and textual revisions satisfactorily address the Reviewer's comment.

3. The neurosteroid argument relies entirely on AMG, which blocks cytochrome P450scc. Ideally, testing a specific neuromodulatory candidate would significantly strengthen the

claim. If the authors do not have candidate neuromodulators, a more detailed discussion of other potential effects of cytochrome P450 inhibition would be useful.

We thank the Reviewer for such a constructive comment. For this reason, in addition to using AMG to inhibit P450scc, we also tested a well-established neurosteroid candidate, allopregnanolone (ALLO), which is known to robustly enhance extrasynaptic GABA receptor function (Br J Pharmacol. 2017; 174:3226-3241). Indeed, exogenous ALLO significantly potentiated the tonic current, indicating that extrasynaptic GABA receptors in our preparation are sensitive to this neurosteroid and ALLO *per se* can mimic the effect of the depolarisation on the tonic current.

We next examined whether ALLO could potentiate the depolarisation-induced increase in tonic inhibition in a within-cell design. ALLO did not enhance I_{GABA} PD relative to ACSF. Although this might suggest that ALLO is not the endogenous mediator responsible for this form of plasticity, it is equally consistent with an occlusion scenario in which depolarisation already recruits the full neurosteroid-sensitive component of tonic inhibition.

Importantly, the Reviewer's comment prompted us to perform an additional experiment that we believe substantially strengthens the manuscript. Specifically, we tested whether the effect of exogenous ALLO application on I_{tonic} was sensitive to gabazine. We performed this experiment because, unlike bicuculline, gabazine did not unmask any basal tonic conductance under control conditions. Indeed, when ALLO was applied, gabazine revealed a clear tonic current. This demonstrates that neurosteroid-dependent modulation of extrasynaptic $GABA_A$ receptors is sufficient to generate a detectable gabazine-sensitive tonic conductance. Notably, I_{GABA} PD is also gabazine-sensitive, supporting the possibility that neurosteroids contribute to the depolarisation-evoked component.

We hope that the inclusion of the ALLO experiments satisfactorily addresses the Reviewer's suggestion and provide a more comprehensive mechanistic framework.

4. The authors bring up astrocytes and microglia as possible sources of GABA in the results section, but do not directly investigate a link. I suggest moving this to the discussion section since it is largely an untested hypothesis.

We fully agree that the suggestion regarding astrocytes and microglia represents a speculative extension of our findings, and we appreciate the Reviewer's recommendation to avoid introducing untested hypotheses in the Results section. In accordance with this comment, we have moved this consideration to the Discussion, where it is now presented explicitly as an untested hypothesis. We are grateful for this suggestion, which has improved the clarity and structure of the manuscript.

Minor:

1. Please refer to your holding current calculation as something other than Ih as for most people, the latter refers to the hyperpolarization-activated h-current.

We agree that referring to the holding current as I_h could be misleading. In accordance with the Reviewer's suggestion, we have replaced this terminology throughout the manuscript, using I_{hold} for the holding current and ΔI_{hold} for its depolarisation-evoked change.

2. There are several grammatical errors and typos to be corrected throughout the manuscript.

We have carefully revised the entire manuscript to correct grammatical errors, typos, and stylistic inconsistencies. This thorough editing step has improved clarity, readability, and overall presentation.

Referee #2

This manuscript reports that tonic GABA inhibition in cortical neurons can be transiently increased by a mechanism that requires endocannabinoid signaling and neurosteroid synthesis. This modulation of tonic inhibition is not accounted for by increased extracellular GABA concentration. This is an interesting and original finding and potentially represents a worthwhile contribution to the existing literature. However, there are several methodological and substantive points raised below that should be addressed to support the validity of the conclusions.

We thank the Reviewer for the positive assessment of the novelty and relevance of our findings, and we appreciate the constructive comments and suggestions, which have helped us further strengthen the methodological clarity and the robustness of our conclusions. Below, we address each point raised by the Reviewer in a point-by-point manner and indicate the corresponding changes implemented in the revised manuscript.

1 - It is reported that recordings were performed on layer 2/3 principal neurons. Does that mean pyramidal neurons, and how were these neurons identified?

We have clarified this point in the Materials and Methods section. Whole-cell patch-clamp recordings were obtained from layer 2/3 pyramidal neurons of SI, visually identified under IR-DIC video microscopy (Zeiss Axioscope FS2, 40× water-immersion objective) based on their characteristic pyramidal-shaped soma and the presence of a clearly visible apical dendrite.

2 - What was the final concentration of DMSO when drugs were applied to the slices, and was a DMSO control performed?

We are sorry we were not clear enough clear in the submitted manuscript. We have updated the Materials and Methods section accordingly. Across all experiments, drugs that required DSMO as solvent were prepared as concentrated stock solutions in 100% DMSO and diluted into 250 mL of recording ACSF to obtain the desired final concentrations. Depending on the volume of stock solution required, the final DMSO content in the bath ranged from approximately 0.04% v/v to a maximum of ~0.19% v/v. Control ACSF solutions were

supplemented with the same DMSO concentration used in the corresponding drug condition to ensure proper vehicle matching.

3 - Bicuculline was used to block GABA-A receptors to quantify tonic inhibition, however bicuculline is also known to block Ca-activated potassium channels, which could confound those measurements. Gabazine (SR-95531) is a widely available selective antagonist at GABA-A receptors that avoids this problem. At least some of the foundational experiments in the manuscript should be repeated with GABAzine to rule out effects on Ca-activated potassium channels.

We thank the Reviewer for raising this important methodological concern, which prompted us to perform additional experiments that we believe substantially strengthen the manuscript.

To directly address this issue, as suggested by the Reviewer, we repeated the foundational experiments using gabazine (SR-95531). Interestingly, while bicuculline consistently revealed a basal tonic current, gabazine failed to uncover any basal tonic conductance, indicating that the bicuculline-sensitive basal current may not reflect a genuine GABAergic tone. Importantly, this also suggests that ambient GABA levels under basal conditions are insufficient to activate extrasynaptic $GABA_A$ receptors to a degree detectable with gabazine at a saturating concentration (100 µM).

Nonetheless, the post-depolarisation shift in holding current was indeed significantly blunted by both bicuculline and gabazine, demonstrating that this depolarisation-evoked current is mediated by $GABA_A$ receptors and not by off-target effects of bicuculline on K^+ channels. Moreover, the gabazine-sensitive post-depolarisation current was significantly reduced by the DAGL inhibitor DO34, mirroring the results obtained with bicuculline and confirming that this potentiation requires on-demand 2-AG mobilisation. These experiments directly address the Reviewer's request to repeat key assays with gabazine.

Interestingly, in a different set of experiments where we tested the effect of the neurosteroid allopregnanolone on I_{tonic} and I_{GABA} PD, we found that when allopregnanolone was exogenously applied to the bath, gabazine, previously unable to reveal any basal tonic current, now unmasked a clear tonic current. This result indicates that gabazine is sensitive to neurosteroid-dependent modulation of extrasynaptic $GABA_A$ receptors and supports the idea that depolarisation triggers neurosteroid signaling, since both the depolarisation-induced ΔI_{hold} and the neurosteroid -induced I_{tonic} are sensitive to gabazine.

Overall, we believe that the Reviewer's comment has substantially improved the manuscript, as the additional experiments prompted by this concern provided stronger mechanistic evidence and reinforced the central conclusions of the study.

4 - AM251 is used to block CB1 receptors but also acts as a CB1 inverse agonist. Why did the authors use AM-251 instead of NESS-0327, which is a potent antagonist of CB1 that is more selective for the CB1 receptor over CB2 than AM-251, and it is a purely neutral antagonist that does not act as an inverse agonist. The potential confounds of inverse agonism should be addressed in the Discussion and complementary experiments with NESS-0327 should be considered.

AM251 was initially chosen because it is widely used in slice electrophysiology and reliably blocks CB1-mediated signalling in several electrophysiological preparations. However, we agree with the Reviewer that AM251 may act as a CB1 inverse agonist and that, in principle, inverse agonism could introduce interpretational confounds. Moreover, as part of the broader statistical revision requested by the Reviewer (next point), the effect of AM251 in the between-cell comparison was no longer significant. This further motivated us to perform complementary experiments using NESS-0327. We found that NESS significantly reduced the bicuculline-sensitive potentiation of tonic current while having no effect on basal I_{tonic}. This important experiment demonstrates that the reduction of the post-depolarisation current reflects genuine blockade of CB1 receptor activation.

5 - It is stated in the Methods that "Studies were designed to generate equal-sized groups", however there are several experiments with very unequal n's between groups, e.g. Figs. 2, 3, and 4. This makes it difficult to support some of the conclusions, and probably relates to why data are presented as mean+SD, instead of the more common mean+SEM.

The sentence in the Methods stating that studies were designed to generate equal-sized groups was inaccurate. We apologise and that sentence has now been removed. Regarding data presentation, we report values as mean ± SD in accordance with the journal's explicit recommendation that " Standard Deviation (SD) must be used instead of Standard Error of the Mean (SEM), unless the use of SEM is fully justified and reported alongside confidence intervals."

We have also carefully re-evaluated all statistical analyses in light of the Reviewer's comment:

- Figure 2 was analysed using non-parametric statistics, which do not rely on assumptions of equal sample sizes or homogeneity of variance. Therefore, the unequal n's do not affect the validity of the conclusions.

- As mentioned also in the previous point, Figure 3 exhibited significant variance heterogeneity as confirmed by The Brown–Forsythe test ($F_{(2, 10.56)} = 7.740$, $p = 0.009$). Thus, Welch's ANOVA was applied, yielding a statistically significant difference ($W_{(2, 14.15)} = 8.292$, $p = 0.004$). Post hoc testing with Dunnett's T3 showed that DO34 remained significantly different from ACSF ($p = 0.012$), whereas AM251 was no longer significantly different from ACSF ($p = 0.066$). The statistics are now updated in the main text.

Figure 4 did not show evidence of variance heterogeneity. The Brown–Forsythe test was non-significant ($F_{(2, 26)} = 1.295$, $p = 0.291$), and Bartlett's test likewise indicated no significant variance differences ($\chi^2 = 4.619$, $p = 0.099$). As the assumptions for standard ANOVA were satisfied, the original statistical approach was retained. The method and result sections have been updated accordingly.

We believe that these revisions ensure that the statistical approach is fully appropriate for each dataset and that the overall conclusions of the study remain well supported.

6 - There is no positive control for the negative result reported for the GAT-1 inhibitor

NO711. Also, could the lack of effect of GAT-1 inhibition represent a ceiling effect?
We thank the reviewer for allowing us to clarify this point. We would like to note that our dataset includes an internal positive control as shown in Fig. 7. Indeed, supplementing the ACSF with 2 μM GABA produced a clear increase in both I_{tonic} and I_{GABA}. These findings indicate that extrasynaptic GABA receptors in our preparation are not saturated following depolarisation, and therefore the absence of an NO711-induced increase in I_{GABA} cannot be attributed to a ceiling effect. Considering this, we believe that the negative result obtained with NO711 is physiologically meaningful and further supports the conclusion that the depolarisation-induced potentiation of tonic inhibition does not arise from an increase in ambient GABA concentration.

7 - The authors should report whether the depolarization protocol also resulted in eCB-mediated DSI. The example sweeps that are shown are too short in duration to observe this, and this is an important point that should be addressed.
We have updated all the figures in the manuscript to include not only the zoomed-in traces but also the full recording sweeps, allowing the reader to clearly visualise the time course of the recording. In addition, we have now performed a quantitative analysis of DSI, as shown in the revised Fig. 1. This analysis demonstrates that the depolarisation protocol reliably induced a transient suppression of inhibitory synaptic transmission in control condition, reflected by significant reductions in sIPSC amplitude (81.68 ± 9.39 %; $p < 0.001$, one-sample t-test), sIPSC frequency (87.41 ± 15.10 %; $p < 0.001$), and sIPSC charge (78.90 ± 11.89 %; $p < 0.001$).

We hope that these additional data and the revised figure satisfactorily address the reviewer's concern regarding the presence of DSI under our experimental conditions.

8 - Example sweeps at expanded time scales are needed to evaluate the quality of the recordings and accuracy of quantification. It is difficult to see whether tonic GABA inhibition was stable during the baseline period, and how it was accurately measured given the high frequency of phasic events. In addition, is the capacitance artifact after depolarization blanked out in the example sweeps? Because the altered tonic current is so brief, it is important to show unedited example sweeps.

We thank the reviewer for this valuable comment. To improve clarity, we have revised the traces accordingly. We have now added expanded-timescale sweeps (5-s duration) together with the full recording trace for the representative examples of basal tonic inhibition.

As also noted in our response to the previous point, we have included the full recording before and after the depolarising step to better illustrate the stability of the baseline holding current. In addition, we have refined the visualisation of the representative zoomed trace before and after the depolarising step. Specifically, the depolarizing step has been partially truncated: the 2-s pre-depolarisation baseline and the 2-s post-depolarisation period are shown in full, whereas only the initial 0.5 s and the final 0.5 s of the 5-s depolarising step are displayed. This presentation allows the baseline and the post-depolarisation shift in holding current to be viewed at an expanded time scale without compromising the integrity of the

recording. As requested, the revised figures also display the capacitive transient following the depolarising step. We also appreciate the reviewer's concern regarding the high frequency of synaptic events and the potential impact on tonic current quantification. To further address this point, we have re-analysed the entire dataset using a more rigorous and conservative procedure. Mean I_{hold} was calculated from 5-s epochs by averaging 10-ms segments sampled every 100 ms, while excluding data points corresponding to IPSC decay. The resulting clean baseline distributions were then quantified using Gaussian fits of the amplitude histograms obtained before and after antagonist application. We believe that this refined analytical approach provides a more accurate and reliable estimate of tonic GABA currents. We hope that these modifications satisfactorily address the reviewer's concerns.

9 - The authors should expand their discussion of the potential functional relevance of the altered tonic inhibition, especially given the extremely brief duration of the effect.
We agree that a more detailed discussion of the potential functional relevance of this transient form of tonic inhibition would strengthen the manuscript. Therefore, in the revised version, we have substantially expanded the Discussion to address how even brief depolarisation-induced increases in GABA receptor–mediated conductance can influence neuronal activity.

Specifically, we highlighted that the combination of a fast retrograde eCB signal with the spatial and temporal precision of intracellular neurosteroid action provides a rapid, reversible tuning of pyramidal cell inhibitory tone. It is reported that even small changes in tonic GABA conductance can propagate to large-scale circuit effects, contributing to anxiety-related behaviours, altering sensory discrimination, or driving pathological hypersynchrony (Botta et al., 2015; Kwak et al., 2020; Cope et al., 2009). Thus, the transient plasticity of tonic GABAergic inhibition described here may provide a mechanism capable of influencing excitability on behaviourally relevant timescales. As an activity-dependent gain-control mechanism, it can transiently suppress excessive firing and pathological hypersynchrony. Moreover, by rapidly restoring a permissive excitability window may support stimulus-dependent forms of plasticity that require precise temporal control of neuronal gain. In addition, by modulating the inhibitory tone of recently active pyramidal neurons, I_{GABA} PD can set the threshold for ensemble recruitment during cortical oscillation, thereby shaping the composition of cortical assemblies.

We are grateful to the Reviewer for highlighting this point, which has helped us clarify the broader implications of our findings.

10 - How was Cm calculated?

Membrane capacitance (Cm) was measured in voltage-clamp mode using a 10 mV depolarizing test pulse delivered from a holding potential of −70 mV. Currents were sampled at 10 kHz, and Cm was calculated from the capacitive transient using the Membrane Test function implemented in pClamp (Molecular Devices). Cm was assessed at the beginning and at the end of each recording.

11 - The manuscript needs to be thoroughly reviewed for grammatical errors, language errors, and numerous typos.

We have carefully revised the entire manuscript to correct grammatical errors, typos, and stylistic inconsistencies. This thorough editing step has improved clarity, readability, and overall presentation. We are grateful to the Reviewer for drawing our attention to this point.

Dear Professor Conti,

Re: JP-RP-2026-289183R1 "**The endogenous cannabinoid system gates plasticity of tonic GABA inhibition**" by Roberto Colangeli and Fiorenzo Conti

We are pleased to tell you that your paper has been accepted for publication in The Journal of Physiology.

The last Word (or similar) version of the manuscript provided will be used by the Production Editor to prepare your proof. When this is ready you will receive an email containing a link to Wiley's Online Proofing System. The proof should be thoroughly checked and corrected as promptly as possible.

Authors should note that it is too late at this point to offer corrections prior to proofreading. Major corrections at proof stage, such as changes to figures, will be referred to the Editors for approval before they can be incorporated. Only minor changes, such as to style and consistency, should be made at proof stage. Changes that need to be made after proof stage will usually require a formal correction notice.

All queries at proof stage should be sent to: TJP@wiley.com.

Following copyediting and typesetting the accepted version will be published online.

Yours sincerely,

Nathan Schoppa
Senior Editor
The Journal of Physiology

IMPORTANT POINTS TO NOTE FOLLOWING ACCEPTANCE OF YOUR PAPER:

• IMPORTANT NOTICE ABOUT OPEN ACCESS: To assist authors whose funding agencies mandate immediate public access to published research findings, The Journal of Physiology allows authors to pay an Open Access (OA) fee to have their papers made freely available immediately on publication.

The Corresponding Author will receive an email from Wiley with details on how to register or log in to Wiley Authors where you will be able to place an order.

• You can check if your funder or institution has a Wiley Open Access Account here:
https://authors.wiley.com/author-resources/Journal-Authors/open-access/author-compliance-tool.html

• TRANSPARENT PEER REVIEW POLICY: To improve the transparency of its peer review process, The Journal of Physiology publishes online as supporting information the peer review history of all articles accepted for publication. Readers will have access to decision letters, including Editors' comments and referee reports, for each version of the manuscript as well as any author responses to peer review comments. Referees can decide whether or not they wish to be named on the peer review history document.

• You can help your research get the attention it deserves! Check out Wiley's free Promotion Guide for best-practice recommendations for promoting your work at: www.wileyauthors.com/eeo/guide. You can learn more about Wiley Editing Services which offers professional video, design, and writing services to create shareable video abstracts, infographics, conference posters, lay summaries, and research news stories for your research at: www.wileyauthors.com/eeo/promotion.

• If you would like to receive our 'Research Roundup', a monthly newsletter highlighting the cutting-edge research published in The Physiological Society's family of journals (The Journal of Physiology, Experimental Physiology, Physiological Reports, The Journal of Nutritional Physiology and The Journal of Precision Medicine: Health and Disease), please click this link, fill in your name and email address and select 'Research Roundup':
https://www.physoc.org/journals-and-media/membernews

EDITOR COMMENTS

Thank you for submitting your revised manuscript to Journal of Physiology. It has been looked at by the two reviewers of the original manuscript and both were quite pleased with the thorough revisions that were made that included the addition of a

number of new experiments, analyses, and changes in the text. They also both cited the importance and novelty of the work. I also appreciate the thorough revisions, including the fact that, in some cases, the authors took the extra step of performing new experiments to make their results more convincing rather than just tone down their conclusions. This has resulted in a stronger manuscript. Congratulations on an excellent study!

REFEREE COMMENTS

Referee #1:

The authors convincingly revised their ms. I have no further concerns.

Referee #2:

The authors should be commended for their very thorough job in addressing all the reviewer and editorial comments. The revised manuscript is significantly improved and represents an important contribution to the field.

****
